# Network analysis of serotonin CNVs shows biological convergence from genetic heterogeneity and discriminates between autism and developmental delay

**DOI:** 10.1038/s41598-026-54264-y

**Published:** 2026-05-26

**Authors:** André Santos, Francisco Caramelo, Joana Barbosa Melo, Miguel Castelo-Branco

**Affiliations:** 1 Institute for Physiology, Faculty of Medicine, Coimbra Institute for Biomedical Imaging and Translational Research (CIBIT), ICNAS, University of Coimbra, 3000-548 Coimbra, Portugal; 2 CIBB, iCBR, Faculty of Medicine, University of Coimbra, 3000-548 Coimbra, Portugal

**Keywords:** Autism spectrum disorder, Global Developmental delay, Genetics, Serotonin, Dopamine, Complex networks, Machine learning, Differential diagnosis, Computational biology and bioinformatics, Genetics, Neuroscience

## Abstract

Differentiating autism spectrum disorder (ASD) from developmental delay (DD) is critical for guiding early intervention, but overlapping features and shared biological mechanisms pose challenges. This study investigates whether copy number variations (CNVs) affecting serotonergic genes carry sufficient information to distinguish between these neurodevelopmental disorders (NDDs). Using network mapping and machine learning, we applied gene ontology (GO) terms related to serotonergic systems to filter CNVs and construct networks modeling genetic and biological variation in ASD and DD. We identified hub nodes and subnetworks reflecting distinct patterns in gene and GO term interactions. ASD networks analysis yielded six genetic clusters, five of which remarkably contained genes specifically linked to serotonergic receptor mechanisms. In contrast, DD networks exhibited greater genetic homogeneity, with just two clusters sharing serotonergic mechanisms. Random Forest classifiers using serotonergic gene features achieved an average prediction accuracy of 85.6%, increasing to 88.6% when combined with dopaminergic dosage features, consistent with the two systems capturing partially non-overlapping biological signal. GO-based features yielded comparable accuracy with fewer inputs, emphasizing their efficiency. These findings demonstrate that different genetic alterations may be associated with disruption of shared biological pathways, each leaving a distinct signature tied to clinical diagnoses. Importantly, although genetic heterogeneity is observed in ASD, we found a homogeneity of serotonergic biological terms, suggesting convergence of mechanisms, which was distinct for each condition. Together, these results suggest that serotonergic CNVs carry discriminatory information for ASD vs. DD classification, and that combining serotonergic and dopaminergic features captures partially non-overlapping biological signal.

**Supplementary Information:**

The online version contains supplementary material available at 10.1038/s41598-026-54264-y.

## Introduction

Children with developmental delay (DD) and autism spectrum disorder (ASD) share overlapping features, such as delays in developmental milestones and communication challenges, which complicate early differential diagnosis^1,2^. However, these conditions also exhibit key distinctions. DD typically involves delays across multiple domains, such as language, problem-solving, and motor skills while ASD is characterized by more persistent deficits in social interaction and communication, as well as restricted and repetitive behaviors.

The neurobiological mechanisms underlying DD and ASD are not fully understood, but disruptions in serotonergic neurotransmission may represent a common factor^3–5^. Serotonin (5-HT), a critical neurotransmitter in both central (CNS) and peripheral nervous systems (PNS)^6,7^, regulates mood, cognition, and social behavior^8–12^. Alterations in serotonin systems have been linked to atypical brain development, consistent with the developmental delays observed in DD and the social and behavioral deficits in ASD^13–15^. Elevated whole-blood serotonin levels, commonly observed in children with ASD, suggest dysregulation in serotonergic systems^16^. Genetic studies also implicate serotonin-related genes, such as serotonin transporter (5-HTT) and receptor (5-HTR) genes, in increased ASD risk^17–21^. Although allele-level associations of specific serotonergic genes have not consistently replicated in recent large-scale exome sequencing studies^22,23^, the physiological evidence for serotonergic dysregulation in ASD—spanning blood biomarkers, developmental biology, and pharmacology—remains robust and motivates a pathway-level rather than allele-level analysis. This study makes no claim that serotonergic genes are more aetiologically important than other high-confidence NDD genes; rather, we test whether a biologically coherent system with documented physiological dysregulation carries sufficient information for classification. Similarly, animal models of DD indicate that disruptions in serotonergic signaling during critical periods have been associated with delays in the acquisition of motor, cognitive, and social skills^24–26^.

Environmental factors may further influence serotonin-related pathways. Maternal exposure to selective serotonin reuptake inhibitors (SSRIs) during pregnancy has been associated with an increased risk of both DD and ASD^13,14^. These associations are consistent with a role for serotonergic function in shaping the neural circuits underlying social, cognitive, and behavioural development.

Beyond serotonin, the interplay between serotonergic and dopaminergic systems significantly influences neurodevelopment, neural information processing and various behavioral aspects^27,28^. Dopamine (DA) is primarily produced in the ventral tegmental area (VTA) and substantia nigra pars compacta (SNc)^29^, where it modulates reward processing, motor control, pain and learning^30–32^. The overlap between serotonergic and dopaminergic pathways—including shared brain regions and receptor interactions—highlights a complex network that regulates sensory processing and behavior^27,33^. Joint dysregulation in these systems has been proposed to contribute to the overlapping clinical profiles of DD and ASD.

Addressing these complexities requires a detailed understanding of how serotonergic and dopaminergic systems interact. This knowledge is crucial for improving early differential diagnoses and developing tailored interventions for these neurodevelopmental disorders (NDDs)^34,35^.

Genetic research presents challenges in uncovering the causes of NDDs due to the rarity and small effect sizes of individual genetic alterations^36^. Additionally, the heterogeneity of these conditions complicates the identification of common mechanisms^37^. To address these issues, complex network analyses that map interactions among genes, proteins, molecules, and disease phenotypes have gained prominence^38^. This approach views biological components as interconnected nodes within networks, where disruptions in functional modules can lead to disease. Analyzing these networks’ topological properties, such as identifying hub nodes, provides insights into the mechanisms underlying conditions like DD and ASD.

The present study asks a specific and testable question: do Copy Number Variations (CNVs) affecting serotonergic genes carry sufficient information to classify ASD versus DD in a network-based machine learning framework? This is not a gene discovery study; it uses Gene Ontology (GO) annotations—a functional classification system—to define a biologically coherent pathway filter, distinct from classical candidate gene association studies that tested specific alleles in case/control cohorts. To address this question, we apply network-based methodologies to map interactions among genes linked with serotonergic pathways in individuals with DD and ASD. By analyzing duplicated and deleted genes and their associated biological mechanisms using GO terminology^39,40^, we classify participants based on gene and GO interactions to predict differential diagnoses. Chromosomal Microarray Analysis (CMA), a widely available supporting diagnostic tool^41^, provides the foundational CNV data. While studies using CMA primarily report statistically significant CNVs, it has potential for data reuse within the scope of network-based methodologies, making it a valuable resource for NDD research.

Our approach integrates complex network analysis and machine learning to address the inherent complexity and heterogeneity of DD and ASD to characterize the topology of serotonergic CNV networks separately for each diagnosis group. Additionally, we integrate these results with our previous work on the dopaminergic differences distinguishing ASD from DD^42^, providing a more comprehensive understanding of the interplay between serotonergic and dopaminergic systems in these conditions and respective diagnostic classification. We further test whether adding dopaminergic features to the serotonergic model significantly improves classification, providing a direct within-cohort comparison of the discriminatory value of each neurotransmitter system.

## Materials and methods

### Data and participants

With the QuickGO API^43^ we created a set of 58 gene ontology (GO)^39,40^ terms related to serotonergic neurotransmission mechanisms, spanning all three GO namespaces (Biological Process, Molecular Function, Cellular Component). These terms were used to identify a set of 60 genes from CNVs in the public dataset curated by the SFARI Gene CNV module^44^. The genes within each CNV region were identified using the BioMart API (Ensembl release 98);^45,46^. a gene was considered affected by a CNV if any coordinate overlap between the CNV region and the gene’s genomic coordinates was present, following coordinate mapping to the GRCh38/hg38 reference genome via Liftover. The 60 serotonergic genes and 58 GO terms used in this study are listed in Supplementary Information (Tables S10 and S11).

A CNV was defined as a genomic segment present in a number of copies deviating from the diploid state. No minimum size or population-frequency filters were applied; triplications and multi-copy duplications were excluded. For this study, we included participants from the SFARI Gene CNV module who had at least one deletion or duplication overlapping one or more of the 60 serotonergic genes (55.7% of CNV–patient pairs were duplications, 44.3% deletions). We only included participants with a single clinical diagnosis in the analysis. Since no new data were acquired, no additional ethical approvals were necessary.

Overall, we selected 409 participants with a diagnosis of ASD (Male: 165; Female: 32; Not Specified: 212) and 408 participants with a diagnosis of developmental delay (DD) (Male: 25; Female: 26; Not Specified: 357). Sex was not used as a covariate because it was unrecorded for 51.8% of ASD participants and 87.5% of DD participants. Age at diagnosis was available for only 100 of 409 ASD participants (24.4%; range 4–31 years, mean 10.9) and for none of the DD participants. Family structure (simplex vs. multiplex) was available for 49.6% of ASD participants (165 simplex, 38 multiplex) but for fewer than 2% of DD participants (Supplementary Information Tables S1–S4). These constraints precluded the use of any of these variables as covariates. The raw data used to initiate this study, as well as the data and code utilized to construct the networks and perform the machine learning analysis, are available in this public repository 10.7910/DVN/HO1JLJ^47^.

### Building networks of participant’s genomic features and diagnostics

Next, we built a network using participants and gene alterations as nodes^48^. In this network, referred to as the Diagnosis-Gene Network, each participant was linked to a gene if a duplication or deletion was present. Subsequently, we constructed a network using GO terms and participants as nodes (Diagnosis-GO Network) by replacing each gene with the associated GO terms. All networks were built using the NetworkX package (version 2.4)^49^ based on the full participant dataset, without sub-sampling. The layout, designed to show a spatial arrangement of all nodes and their links, was rendered using Gephi software^50^.

Node degree (the number of links attached to a node) was used to size nodes, and colors were used to differentiate between various types of nodes. This approach helped to identify network hubs—nodes with a high degree in a network predominantly characterized by nodes with a low degree^51^. The Fruchterman–Reingold (FR) algorithm^52^ was employed to organize the nodes using a gravity-based approach, where the higher the degree of a node, the stronger the force it exerted to attract linked nodes and repel unlinked ones.

To enable visual comparisons, the parameters used in the FR algorithm—including the display area for the nodes, the gravity force, and the speed at which changes occurred until the algorithm stabilized—were set equally for all networks.

### Network analysis methods and similarity networks

From the network topologies we extracted standard centrality and topological measures^53^: number of nodes, number of edges, average degree, network density, diameter, and radius.

Next, we analyzed the hubs of each network in terms of their degree and the average degree of their neighbors^54^. These measures help to understand the importance of the hub in the network structure.

Consequently, we performed the network analysis by addressing subnetworks of genes shared by participants within the same diagnosis and the GO terms linked to these genes. First, two subgraphs were projected (one for each diagnosis) using participant diagnosis and their links to serotonergic genes. Then, using the generalized similarity^55^ algorithm, we identified groups of genes frequently shared among participants of a particular diagnosis. Finally, we added the GO terms linked to these groups of genes. This type of knowledge helps not only to compare differences between clusters of biological mechanisms related to ASD and DD diagnostics but also to collate differences between participants within the same diagnostic category.

### Classification of type of diagnosis using machine learning

**Fig. 1:**
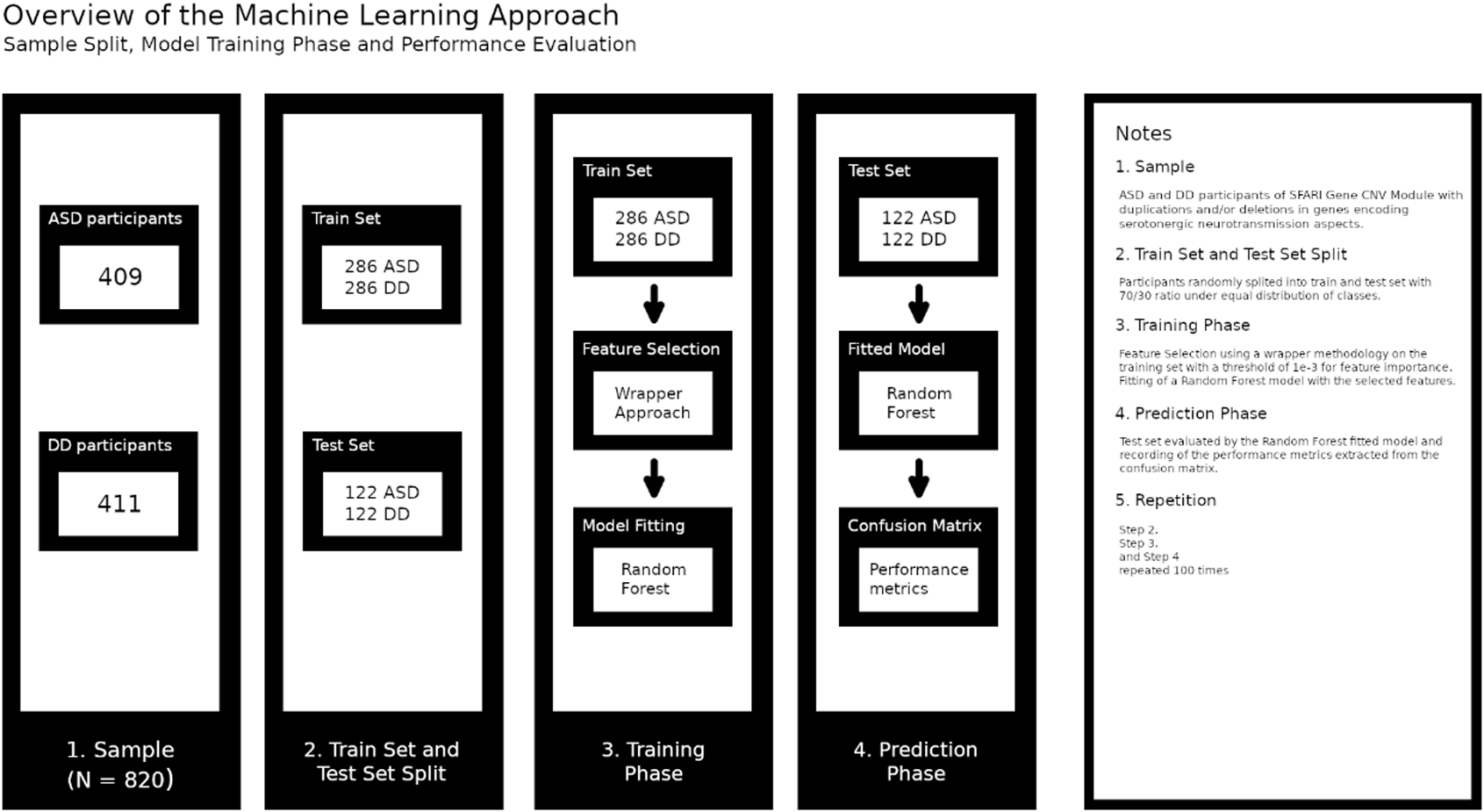
Overview of the Machine Learning approach. The sample includes ASD and DD participants of the SFARI Gene CNV Module with duplications and/or deletions in genes encoding serotonergic neurotransmission aspects. This sample was randomly split into train and test sets with a 70/30 ratio under equal distribution of classes. In the training phase, a feature reduction using a wrapper approach operating with a Random Forest algorithm was performed on the training set. Next, the training set was used to train a Random Forest algorithm. The test set was then assessed by the Random Forest algorithm and the classifier performance was recorded. This process (steps 2, 3, and 4) was repeated 100 times.

**Fig. 2:**
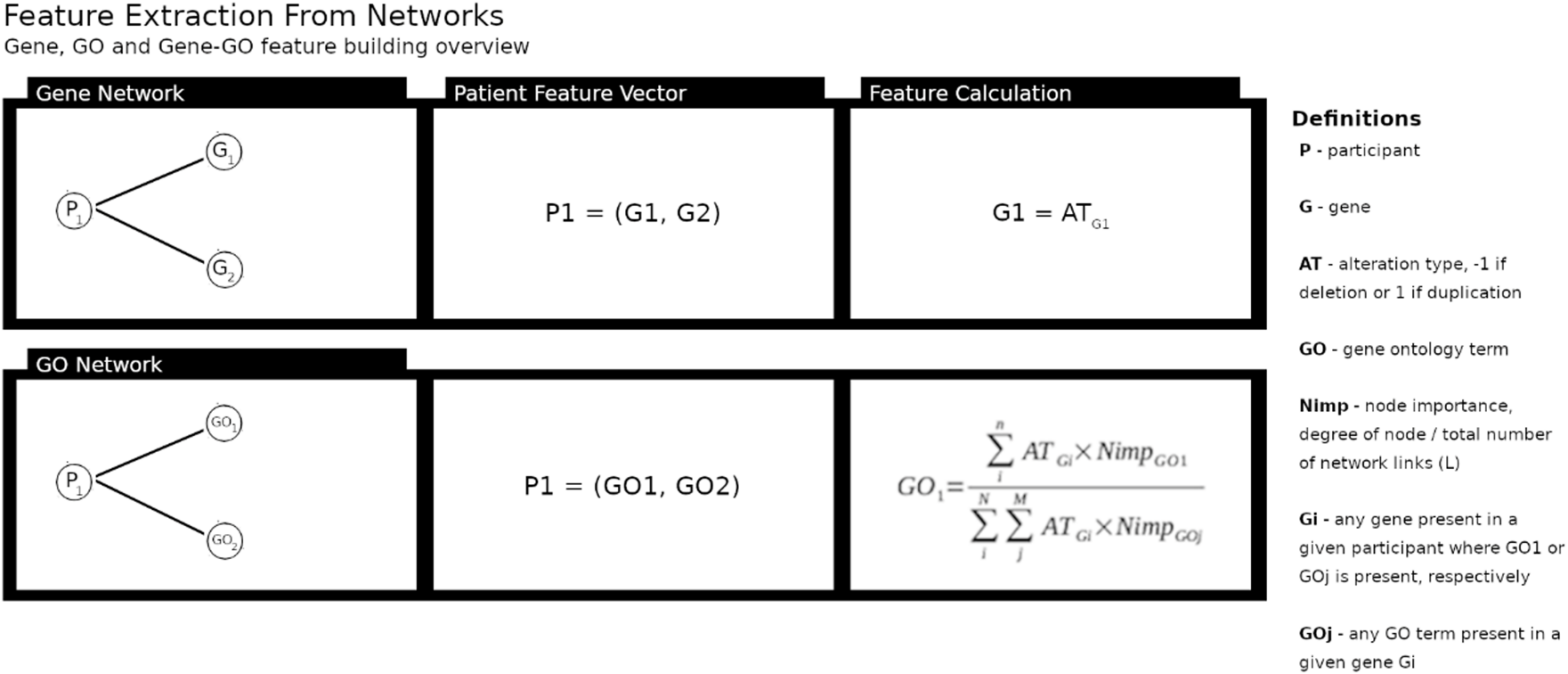
Example of feature extraction from the networks. In the Gene Network, the vector of features is given by multiplying each gene node’s importance by the type of alteration (1 for duplications or − 1 for deletions) that links a participant to a gene. In the GO Network, the same principle was applied to build the features vector; when a GO term was shared by multiple genes its product was summed.

In this step, we used relations present in the Diagnosis-Gene Network and Diagnosis-GO Network, extracted participant features with a single pass feature extraction, and trained machine learning classifiers to predict the participant’s diagnosis^56^. Figure 1 provides an overview of the ML approach. The features used to train the algorithm were selected from the training sample and consist of: 1) unit vectors that represent the presence of links between a participant and a genetic alteration (e.g., 1 for duplication, -1 for deletion, and 0 for no alteration) in the case of the Diagnosis-Gene Network or; 2) weighted vectors of the frequency of links between participants and the GO terms attached to their genetic alterations in the case of the Diagnosis-GO Network (Fig. 2).

To build the features, we measured the importance of each non-participant node (gene node or GO term node) by dividing the node degree (the number of other nodes attached to it) by the total number of links. Then, for each participant, if they were linked to a particular node, we used its node importance multiplied by the type of the participant gene dosage (Fig. 2). For example, in the gene network, each gene was a feature. The value for each feature is given by the node degree over the total number of network links, which we denoted as network node importance, multiplied by a factor of 1 or -1 representing the participant CNV type—a duplication or deletion, respectively. The gene ontology network was framed in the same way.

Thus, we generated two datasets that described the serotonergic imbalances of the participants: one related to genetic variation and another related to GO term occurrences. Using the same approach, we generated two additional datasets: one with gene features and another with GO term features, incorporating both serotonergic and dopaminergic imbalances identified in the participants’ CNV genes.

The SciKit-Learn package^57^ was used to train and test Random Forest classifiers to predict the participants’ diagnoses based on network features and the participants’ type of gene dosage, duplication, or deletion. We started by randomly splitting the participants into a training and a test set, with a 70/30 ratio, ensuring an equal distribution of classes. In the training set, we identified the features with more discriminant power using a wrapper methodology with a threshold of 1 × 10^−3^ for feature importance^58^, which were then used to train the classifier. The test set was validated on the previously trained algorithm, and the confusion matrix was recorded. This process was repeated 100 times for each type of dataset. In the end, we calculated the mean and the standard deviation of several metrics, such as accuracy, precision, and recall, from the confusion matrix obtained at each run^59^. To assess whether the addition of dopaminergic features significantly improved classification, accuracy distributions from the serotonergic-only and combined models were compared across the 100 runs using the Nadeau and Bengio corrected paired t-test and the Wilcoxon signed-rank test; effect sizes were estimated using Cohen’s d.

## Results

### Sample

Data used in this work come from the SFARI Gene CNV Module, which gathers CNV data related to ASD from several studies. In Table 1, the number of participants is categorized by diagnostic categories where duplicated or deleted serotonin-coding genes were present. Given the number of participants without sex specification, we decided not to conduct a sex-specific analysis.

**Table 1:** Distribution of Participant Diagnosis: Diagnostic and the number of participants (N) identified with duplicated or deleted genes containing information related to serotonergic aspects on the SFARI Gene CNV Module.

Diagnosis	Number of Participants (N)	Sex (M/F/NS)
ASD	409	165/32/212
DD	408	25/26/357
Total	817	

The sex of the participants is reported in the Sex column as males (M), females (F), or not specified (NS). Only participants with a single diagnostic category were considered.

### Diagnosis related gene networks

**Fig. 3:**
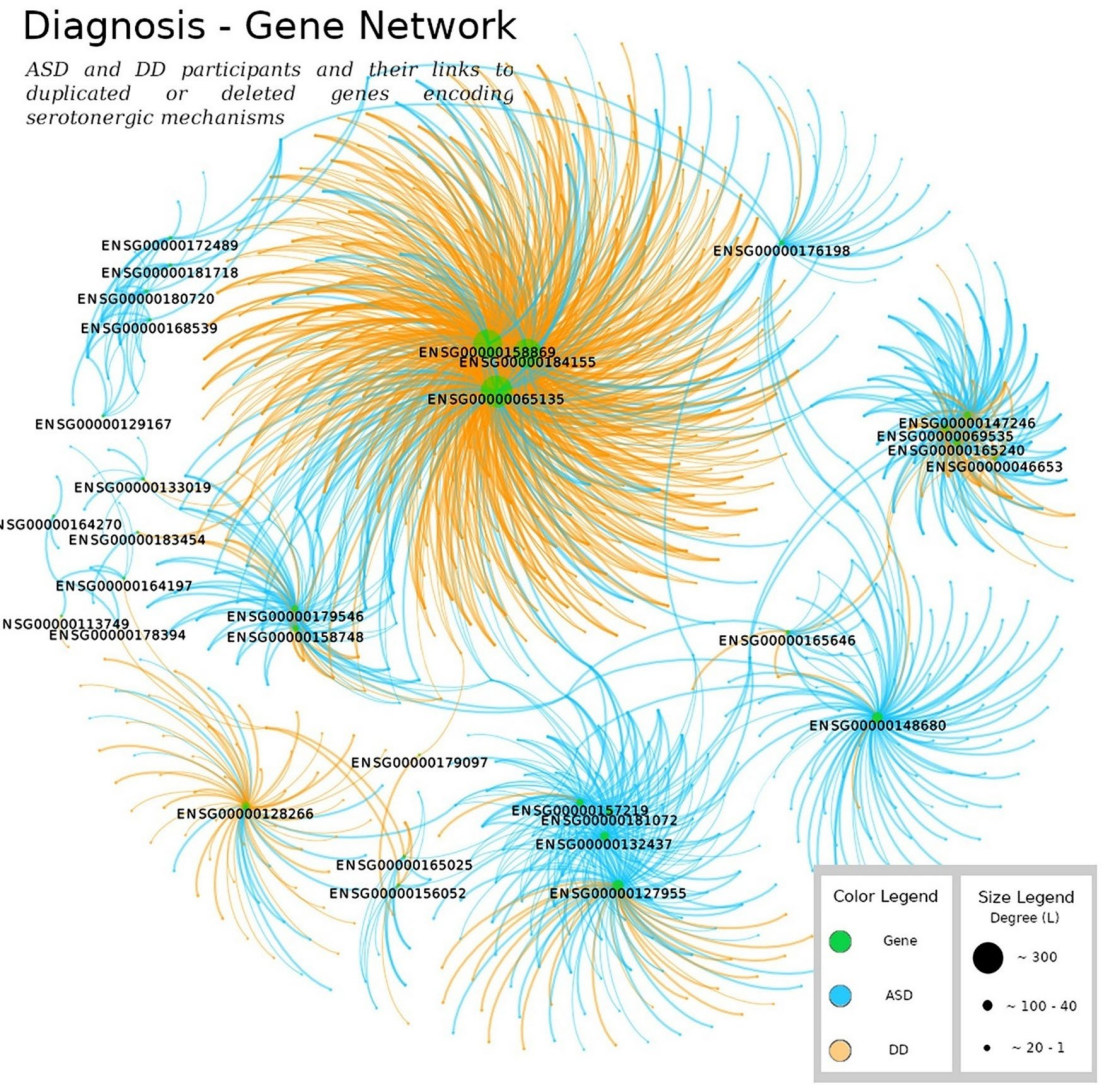
Network of participants diagnosed with ASD or DD and their relationship with deleted or duplicated serotonergic genes. Green nodes represent the serotonergic genes and are labeled with the respective Ensembl unique identifier. Blue lines represent relationships between genes and participants affected by ASD, whereas orange lines represent relationships between genes and participants with DD. The size of a gene’s node (green nodes) is proportional to the number of participants linked to it (ex.: larger gene nodes have more participants attached to it).

The Diagnosis-Gene Network (Fig. 3) represents participants with ASD and DD and their relationship with duplicated or deleted genes related to serotonergic mechanisms. Analysis of the macroscopic characteristics of this projection, such as Degree, reveals insights into which genes are more frequently tied to a diagnosis and the patterns by which a gene or subset of genes is related to a particular diagnosis. Analysis of degree and network topology reveals which genes are most frequently linked to each diagnosis and whether participants cluster around shared alterations or distribute across distinct subsets—and identifies hubs, a small number of highly connected genes that account for most network links.

For the DD diagnosis, Fig. 3 shows that genetic alterations of three genes, ENSG00000065135 (*GNAI3*—G Protein Subunit Alpha I3), ENSG00000158869 (*FCER1G*—Fc Epsilon Receptor Ig), and ENSG00000184155 (*OR10J5*—Olfactory Receptor Family 10 Subfamily J Member 5), are linked to the majority of participants affected by this condition, resulting in one large cluster in the middle of the network. These three genes are also hub genes since they account for almost all connections with DD participants.

Conversely, for the ASD diagnosis, participants are distributed across different subsets of genetic alterations, resulting in several clusters near the network boundaries. This evidence indicates greater genetic heterogeneity among ASD participants than among DD participants in the sample.

Additionally, a small portion of ASD participants share the same three genetic alterations that are predominant in DD cases. This pattern, observed in other small network clusters, suggests genetic overlaps between the two diagnoses.

These network patterns reveal significant genetic differences both between and within the diagnoses, while also highlighting the genetic complexity inherent in these two conditions.

### Diagnosis related gene ontology (GO) networks

**Fig. 4:**
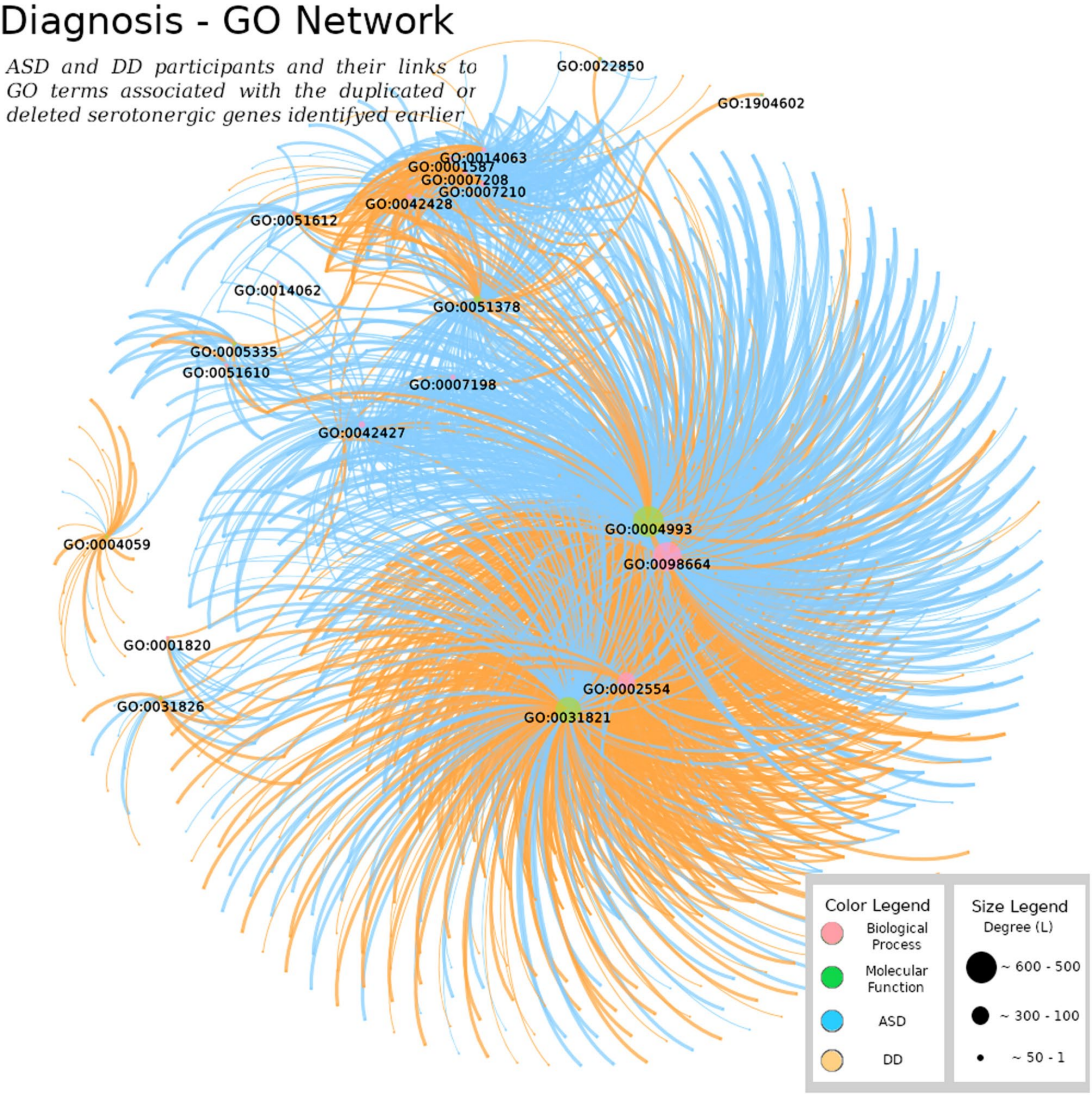
Network of participants diagnosed with ASD or DD and their relationship with serotonergic GO terms linked to their deleted or duplicated serotonergic genes. Pink nodes represent GO serotonergic biologic processes and green nodes mark GO serotonergic molecular functions. Both GO nodes are labeled with the respective GO unique identifier. Blue lines represent relationships between GO terms and participants affected by ASD, whereas orange lines represent relationships between GO terms and participants with DD. The size of a GO term node (pink and green nodes) is proportional to the number of participants linked to it (ex.: larger GO term nodes have more participants attached to it).

The primary purpose of the Diagnosis-GO Network (Fig. 4) is to illustrate the relationships between participants with ASD or DD and the serotonergic GO terms (biological processes and molecular functions) linked to their deleted or duplicated serotonergic genes. This network can reveal which GO terms are more frequently associated with each diagnosis, the relationships between these GO terms and the two types of diagnosis, and whether any GO terms function as network hubs.

Analysis of Fig. 4 shows that most participants are linked to a small set of GO terms. Participants with ASD are primarily associated with GO:0004993 (G protein-coupled serotonin receptor activity) and GO:0098664 (G protein-coupled serotonin receptor signaling pathway), whereas participants with DD are frequently associated with GO:0031821 (G protein-coupled serotonin receptor binding) and GO:0002554 (serotonin secretion by platelet). However, there is some overlap between these GO terms and both diagnoses, indicating they are not exclusive to either condition.

Figure 4 also confirms that these four GO terms are network hubs, as they connect with most participants from both diagnoses. This contrasts with Fig. 3, where no hubs were identified among ASD participants due to their distribution across different genetic clusters.

Near the network boundaries, clusters of GO terms encompass the remaining participants. These clusters differ from the peripheral genetic clusters in Fig. 3, as many participants in a cluster are also linked to the central hub cluster, with only a few participants isolated in peripheral clusters.

These patterns suggest a serotonergic convergence of molecular functions and biological processes underlying the genetic heterogeneity observed in Fig. 3.

### Network analysis

Analyzing network metrics, such as centrality measures and the characteristics of network hubs and their neighborhoods, is a valuable resource to confirm previously observed patterns and uncover new serotonergic network characteristics that might be difficult to detect through macroscopic analysis alone.

Table 2 presents the centrality measures for each network. Considering N (number of nodes) and L (number of links), we identify that the Diagnosis-Gene network has 98 fewer nodes and 875 fewer links than the Diagnosis-GO network. This reduction decreases the average degree (< k >), the density (the quotient between the number of links and the total possible number of links) of the Diagnosis-Gene network, and also impacts its diameter (the maximum distance between any two nodes) and radius (the minimum distance of the maximum distances between pairs of nodes).

**Table 2:** Diagnosis-Gene Network and Diagnosis-GO Network centrality measures.

Network	N	L	< k >	Density	Diameter	Radius
Diagnosis-Gene	740	1748	2.362	0.006	13	7
Diagnosis-GO	838	2623	3.130	0.007	7	4

The total number of nodes (N), the total number of links (L), the density (the total number of links L divided by the total number of possible links in a network with N nodes), the diameter (the maximum path between the two most distant nodes) and the radius (the minimum path between the two most distant nodes).

For example, comparing the densities of the two networks, we observe a difference of 0.001 units. Yet, this slight change significantly impacts the two network structures. The diameter of the Diagnosis-Gene network is 13 nodes, whereas the diameter of the Diagnosis-GO network is 7 nodes. This indicates that the two most distant nodes in the Diagnosis-GO network are almost half as far apart as those in the Diagnosis-Gene network. This suggests the presence of more strongly tied communities (clusters with numerous internal links) in the Diagnosis-GO network compared to the Diagnosis-Gene network. Studying these communities is scientifically valuable as it helps identify key biological mechanisms disrupted in groups of participants, which can be used to meaningfully partition ASD heterogeneity derived from different genetic factors.

Indeed, by comparing Figs. 3 and 4, it is evident that the clusters in Fig. 3 are less strongly tied than those in Fig. 4. Additionally, there is a noticeable global shrinkage (closer proximity between nodes) in the Diagnosis-GO network compared to the Diagnosis-Gene network. This shrinkage results from replacing gene nodes with their corresponding GO terms, and the analysis of the network’s centrality measures supports this observation. Besides impacting the network structures, this also affects the network’s biological meaning. For example, in the Diagnosis-Gene network, genetic heterogeneity is observed among participants with ASD compared to genetic homogeneity among participants with DD. Conversely, in the Diagnosis-GO network, the predominant pattern is a homogeneity of serotonergic terms, differing for each type of diagnosis.

#### Network hubs and neighborhood

Hub analysis quantifies how many participants are linked to major nodes and whether hubs are isolated or embedded in denser clusters (via average neighbour degree).

Table 3 characterizes the hubs of the Diagnosis-Gene network and Diagnosis-GO network by name, degree (k), the average number of neighbors of participant nodes linked to hubs, and, in the case of genetic hubs, their hub GO term counterparts.

**Table 3:** Networks’ maximum degree nodes (hubs): the node, their name, the node degree (k), the average degree of the hubs neighbors, and the direct relation between gene hubs and GO hubs.

Network	Hub	Name	k	Average Neighbour Degree	GO relation
Diagnosis-Gene	ENSG00000065135	GNAI3	334	3.077	GO:0031821
	ENSG00000158869	FCER1G	301	3.156	GO:0002554
	ENSG00000184155	OR10J5	301	3.156	GO:0098664; GO:0004993
Diagnosis-GO	GO:0004993	G protein-coupled serotonin receptor activity	605	3.884	
	GO:0098664	G protein-coupled serotonin receptor signaling pathway	561	3.627	
	GO:0031821	G protein-coupled serotonin receptor binding	491	3.458	
	GO:0002554	Serotonin secretion by platelet	313	4.015	

In the Diagnosis-Gene network, the most connected node is the gene *GNAI3* with 334 connections to participants. The other two identified gene hubs, *FCER1G* and *OR10J5*, each have 301 connections to participants (present in approximately 37% of the participants). Together, these three hubs account for approximately 54% of the connections in this network.

Analyzing the hub’s neighbor degree in the Diagnosis-Gene network, we find that a participant linked to any of these gene hubs is likely to have, on average, three links with other genes in the network. With the help of Fig. 3, it can be detected that these three genes are likely the network gene hubs linked to the majority of participants with DD. In other words, in the Diagnosis-Gene Network, if a participant is linked to one gene hub, it is likely also linked to the other two gene hubs and is likely to be a participant with a DD diagnosis.

Regarding the hubs of the Diagnosis-GO network, we can identify that the serotonergic Gene Ontology (GO) term related to the activity of serotonin receptors attached to G-proteins is the most connected node. This hub links 605 times with different participant nodes, indicating that 74% of the participants have a disruption related to this molecular function. When compared to the most connected hub of the Diagnosis-Gene network (the *GNAI3* gene hub), it has almost twice the number of links (k) than the gene hub. This information complements the results presented earlier in Figs. 3, 4, and Table 2.

The other three hubs in the Diagnosis-GO network represent serotonergic mechanisms related to the signaling, binding, and secretion of serotonin. Interestingly, the hub with the fewest connections (GO:0002554) has only 21 fewer connections than the most connected hub of the Diagnosis-Gene network (*GNAI3*) and 12 more connections than the other two gene hubs. Additionally, the Diagnosis-GO hubs together encompass 75% of the links present in this network.

The average neighbor degree of the Diagnosis-GO hubs shows that a participant linked to one of these hubs has on average, 3–4 links with other GO terms. Considering the information depicted in Fig. 4, it can be detected that the majority of these links are distributed among the GO term hubs identified in Table 3, forming a large central cluster. However, it is also possible to notice some participant connections with other less connected GO terms and one or more of the identified GO hubs, resulting in small peripheral clusters. This contrasts with the Diagnosis-Gene network, where it is rare for participants in peripheral clusters to connect with a gene belonging to the gene hub set.

Finally, the GO relation column of Table 3 identifies the serotonergic GO terms associated with gene hubs, providing further insight into the state of the two diagnoses. For instance, considering the gene hub *OR10J5*, which has 301 connections with participants, it is related to the G protein-coupled serotonin receptor activity GO term, which has 605 connections with participants—more than twice the connections found in the gene hub. This implies that approximately 50% of participants have genetic disruptions targeting the receptors involved in serotonin activity stemming from other genes than the gene hub *OR10J5* itself.

With the help of Fig. 3, we can observe that for participants with DD, disruptions related to G protein-coupled serotonin receptor activity are likely linked to the gene hub *OR10J5* (ENSG00000184155). In contrast, the same disruptions in participants with ASD are likely to arise from a set of genes that also target this molecular function but individually have a smaller number of links compared to the gene hub *OR10J5*.

To characterize network architecture beyond degree-based hubs, extended topological metrics were computed separately for the gene-dosage and GO-term networks (Figs. 3 and 4, respectively) by diagnosis group (Table 4). The ASD gene network shows substantially higher modularity than the DD network (0.61 vs 0.17), with seven communities detected, indicating that serotonergic CNVs in ASD are distributed across multiple functionally distinct gene clusters rather than concentrated in a single connected block. By contrast, the DD network has a markedly higher clustering coefficient (0.92 vs 0.68), reflecting concentrated connectivity around a small number of high-frequency hub genes (*GNAI3*, *FCER1G*, *OR10J5*—each present in over 72% of DD participants) (Check Supplementary Information Tables S7, S8 and, S9). *HTR7* is the dominant hub in the ASD network by both degree and betweenness centrality; global efficiency is comparable across diagnosis groups (range: 0.26–0.42).

**Table 4:** Extended topological metrics of the serotonergic bipartite networks by diagnosis group (ASD vs. DD).

Metric	Gene ASD	Gene DD	GO ASD	GO DD
Average clustering coefficient	0.676	0.924	0.486	0.672
Global efficiency	0.263	0.276	0.413	0.424
Modularity	0.610	0.170	0.237	0.208
Communities	7	9	3	3
Top hub (degree)	HTR7	GNAI3	GPCR serotonin receptor activity	GPCR serotonin receptor binding
Top hub (betweenness)	HTR7	GNAI3	GPCR serotonin receptor activity	GPCR serotonin receptor binding

### Gene generalized similarity networks enhanced with GO terms

The Generalized Similarity algorithm operates on bi-partite networks (networks where nodes of one type only interact with nodes of another type, such as the Diagnosis-Gene and the Diagnosis-GO networks) and facilitates the grouping of nodes with similar connections. For instance, when applied to the Diagnosis-Gene network, the algorithm identifies clusters of genes that are frequently disrupted in groups of participants.

**Fig. 5:**
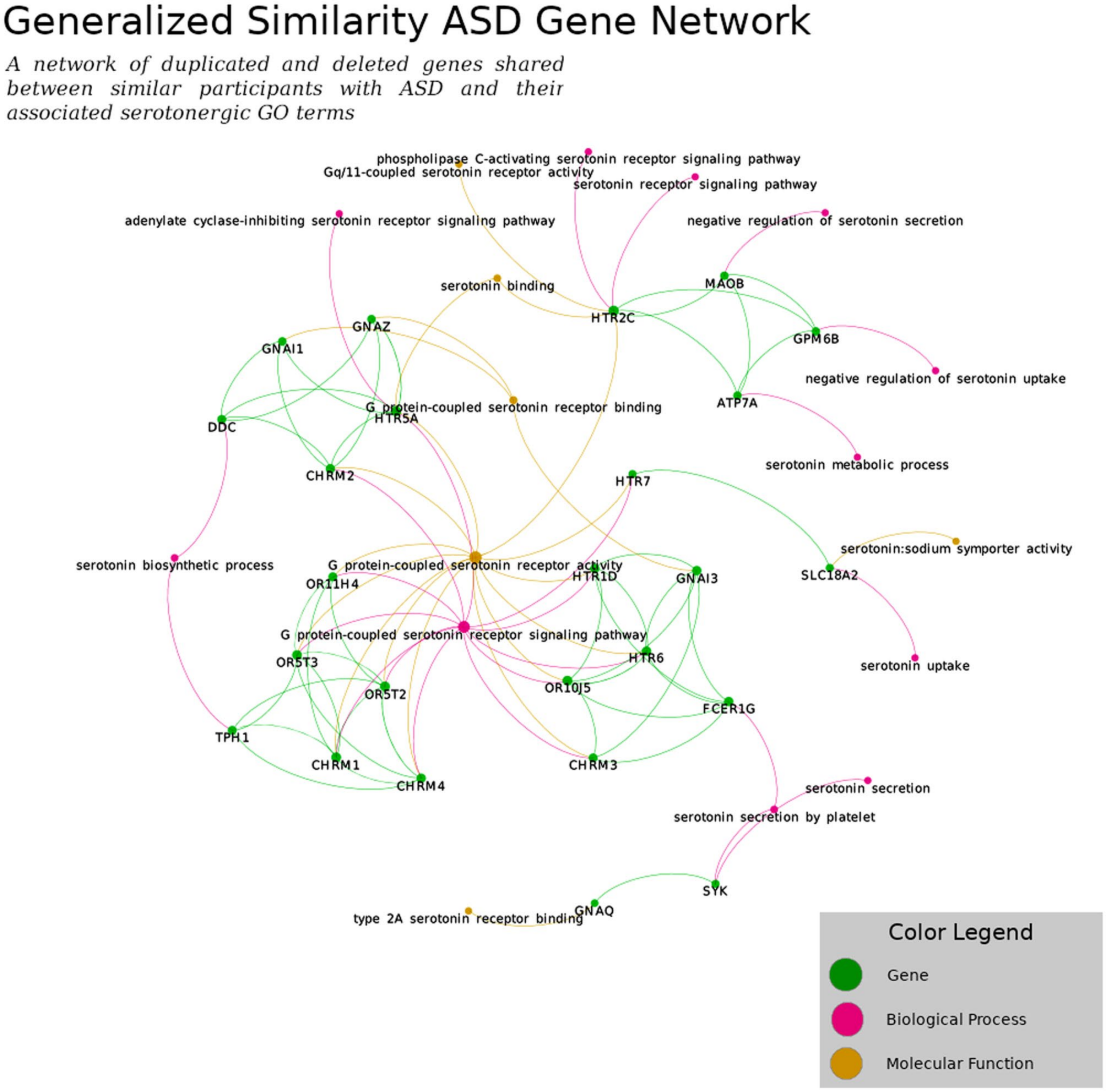
Gene generalized similarity network enhanced with GO terms for ASD participants. Green nodes represent serotonergic genes deleted or duplicated in ASD participants. Pink nodes and orange nodes represent GO terms used for serotonergic biological processes and molecular functions, respectively. Links between green nodes represent gene subsets based on similar genetic variations shared by participants with ASD. Links between GO terms (pink and orange) and genes were added to the graph to show the serotonergic GO term attached to a particular gene.

**Fig. 6:**
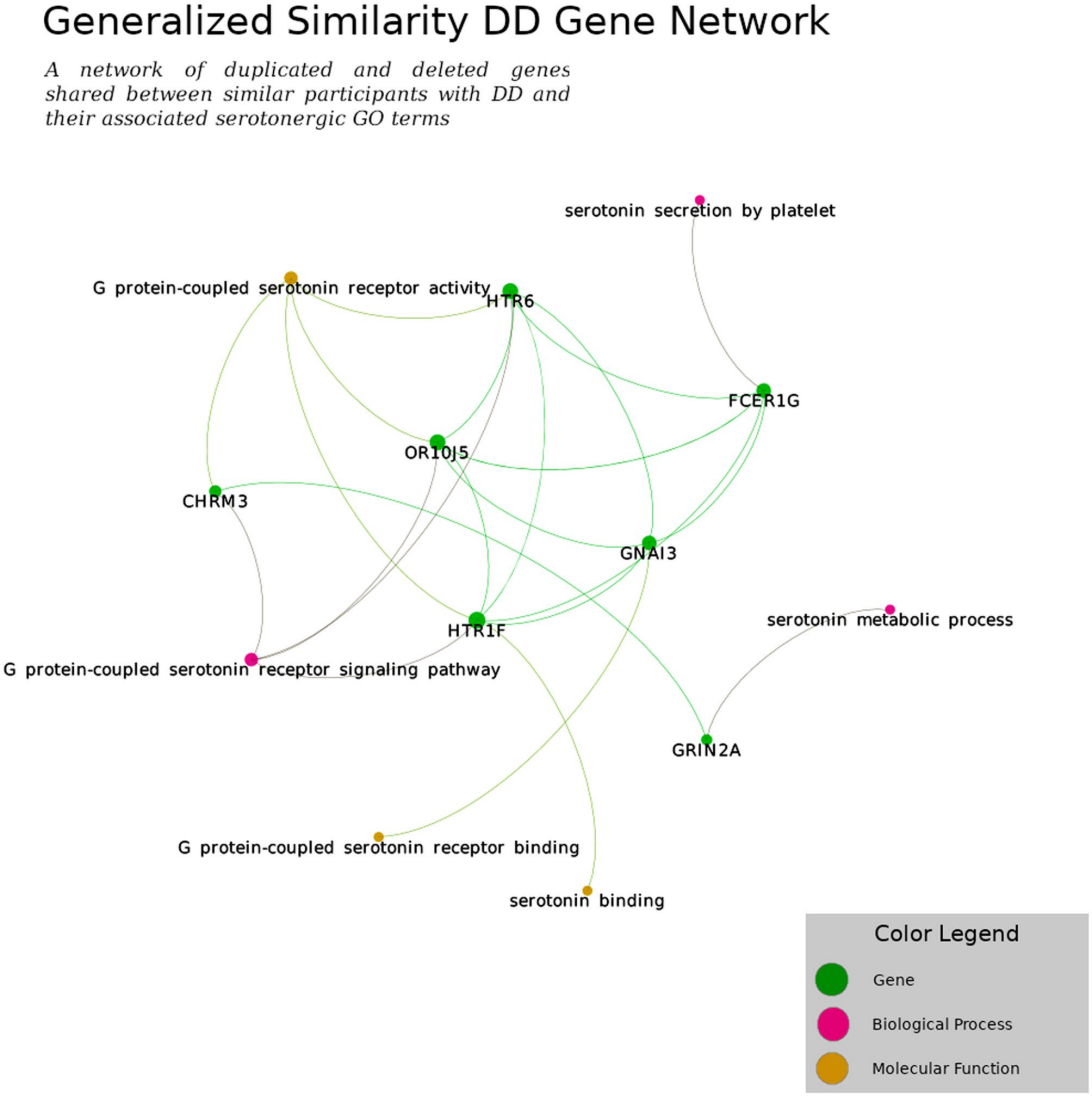
Gene generalized similarity network enhanced with GO terms for DD participants. Green nodes represent serotonergic genes deleted or duplicated in DD participants. Pink nodes and orange nodes represent GO terms used for serotonergic biological processes and molecular functions, respectively. Links between green nodes represent gene subsets based on similar genetic variations shared by participants with DD. Links between GO terms (pink and orange) and genes were added to the graph to show the serotonergic GO term attached to a particular gene.

To better characterize the serotonergic genes involved in the two types of diagnoses, we project a subgraph for each diagnosis—networks where only the participants and genes linked to the diagnosis of interest are projected—and then apply the Generalized Similarity algorithm. This results in two networks that contain genes linked to each other, characterizing groups of participants within each diagnosis (Figs. 5 and 6). Finally, we add the serotonergic GO terms linked to each of the genes identified by the algorithm.

#### ASD Gene-GO network

Six distinct gene clusters were identified for participants with ASD, with the number of genes per cluster ranging from a minimum of two genes to a maximum of six genes. Each gene cluster represents a set of participants with ASD who exhibit similar genetic alterations.

Although these gene clusters are isolated from each other (there are no links between different gene clusters), we verified that in five of them, there is a perturbation of the same serotonergic mechanisms. Specifically, these mechanisms include the molecular function associated with G protein-coupled serotonin receptor activity and the biological process of the G protein-coupled serotonin receptor signaling pathway.

These mechanisms were identified earlier in Table 3 as GO term hubs of the Diagnosis-GO network, which could explain the similarities between participants with ASD from different genetic clusters identified in Fig. 5.

Additionally, we verified that each genetic cluster has a particular set of serotonergic mechanisms that are not shared between different clusters. These unique mechanisms could explain the differences among participants with ASD belonging to different genetic clusters.

#### DD gene-GO network

Figure 6 represents the gene generalized similarity network for participants with DD. In this network, we identified two distinct genetic clusters, one consisting of two genes and another comprising five genes.

The serotonergic mechanism linked to the largest gene cluster includes all the GO term hubs identified in Table 3. This fact, along with the small number of gene clusters identified compared to the generalized gene network for participants with ASD, supports the idea that there is less genetic heterogeneity associated with serotonergic mechanisms in participants with DD.

In the largest gene cluster, we notice an overlap of genes with a gene cluster in Fig. 5, which characterizes participants with ASD. Interestingly, this overlap includes all the genes identified as gene hubs in Table 3 for both diagnoses. However, in the gene cluster associated with the DD diagnosis, the *HTR1F* (5-Hydroxytryptamine Receptor 1F) gene is present (Fig. 6), whereas, in the ASD gene cluster, the *HTR1D* (5-Hydroxytryptamine Receptor 1D) and *CHRM3* (Cholinergic Receptor Muscarinic 3) genes are present instead (Fig. 5). These differences might be potentially useful for differentiating between diagnoses.

### Classification of type of diagnosis using a machine learning approach

To quantify the quality of the information contained in the networks of Figs. 3 and 4, we tested them in a differential diagnosis machine learning classification task. We extended this approach to gene dosage effects of another neurotransmitter, dopamine.

We included participants’ genetic alterations targeting the dopaminergic process and the respective GO terms. We then measured the performance of differential diagnosis classification in this new setup to determine whether adding dopaminergic features enhances classification performance and to which extent.

Table 5 presents the classification results. Overall, algorithms achieved higher accuracy during the test phase with both gene and GO vectors, regardless of using the serotonergic approach alone or combined with the dopaminergic approach.

**Table 5:** Machine Learning Results.

Measure	Dataset							
	Serotonin				Serotonin-Dopamine			
	Diagnosis-Gene		Diagnosis-GO		Diagnosis-Gene		Diagnosis-GO	
	mean	sd	mean	sd	mean	sd	mean	sd
TN (DD)	110	4	105	6	111	3	110	3
FP	12	4	17	6	11	3	12	3
FN	23	4	26	5	17	4	17	4
TP (ASD)	99	4	96	5	105	4	105	4
Train accuracy (%)	89.7	0.7	85.6	0.8	93	0.6	92.9	0.6
Test accuracy (%)	85.6	2.2	82.4	2.1	88.6	1.8	88	1.8
Nº of features	51	3	20	1	111	6	68	2
AUC	0.856	0.022	0.824	0.021	0.886	0.018	0.880	0.018
DD Precision (%)	82.9	2.5	80.5	3	86.8	2.5	86.4	2.4
DD Recall (%)	89.8	3.3	85.9	5	91.1	2.3	90.4	2.3
ASD Precision (PPV %)	89.0	3.2	85.1	3.8	90.7	2.2	90	2.2
ASD Recall (%)	81.4	3.3	78.9	4.5	86	3	85.7	3
DD F1 score (%)	86.2	2.1	83	2.3	88.9	1.7	88.3	1.7
ASD F1 score (%)	85	2.3	81.8	2.2	88.3	1.9	87.7	1.9

TN, true negatives; FP, false positives; FN, false negatives; TP, true positives; AUC, area under the curve.

The maximum test accuracy recorded in the serotonergic approach equals 85.6% ± 2.2% when using gene vectors and required 51 ± 3 features. Using GO terms required fewer features (20 ± 1) but resulted in lower accuracy (82.4% ± 2.1%), approximately 8 fewer correct diagnoses per run.

The same measure for the serotonergic + dopaminergic approach achieved a test accuracy of 88.6% ± 1.8% using gene vectors and used 111 ± 6 features. Using GO terms required fewer features (20 ± 1) with similar accuracy stability (88.0% ± 1.8%), approximately 1 fewer correct diagnosis per run.

Comparing the best test accuracy in the serotonergic + dopaminergic approach with the serotonergic approach alone, we observed a 3% increase, equating to approximately 7 more correct diagnoses per run.

The improvement in GO-term accuracy was statistically significant (Nadeau & Bengio corrected t(99) = 3.05, *p* = 0.003; Cohen’s d = 2.02). For gene-dosage features, the improvement showed a large effect size (Cohen’s d = 0.98), significant by Wilcoxon signed-rank test (*p* < 0.001), though not by the more conservative Nadeau & Bengio correction (*p* = 0.14) (Supplementary Information Table S6). These results indicate that dopaminergic and serotonergic CNVs capture partially non-overlapping biological signal.

To individually analyze the prediction quality of each diagnosis, we verified the precision and recall measures. Overall, the precision for the diagnosis of ASD prediction was slightly higher than for the diagnosis of DD prediction. Using GO vectors, this difference decreased (approximately 4.6%); however, the precision for ASD (85.1% ± 3.8%) and DD (80.5% ± 3%) was still lower compared to using gene vectors.

The precision for both diagnoses was higher with the serotonergic + dopaminergic approach than with the serotonergic approach alone. However, this difference is unlikely to translate into higher clinical performance. For example, the ASD precision with the serotonergic + dopaminergic approach using gene vectors was 90.7% ± 2.2%, whereas the ASD precision with the serotonergic approach and same data type was 89% ± 3.2%. Although the precision was higher with gene vectors than with GO vectors, the differences within the same diagnosis type were much smaller with the serotonergic + dopaminergic approach. Overall, the serotonergic + dopaminergic approach achieved strong PPV for both ASD (gene: 90.7% ± 2.2%; GO: 90% ± 2.2%) and DD (gene: 86.8% ± 2.5%; GO: 86.4% ± 2.4%), consistent with potential utility for differential diagnosis classification of neurodevelopmental disorders.

Recall rates were consistently higher for DD (85.9–91.1%) than ASD (78.9–86%), suggesting superior detection of true DD cases in this cohort. The serotonergic + dopaminergic models achieved near-identical peak recall for both diagnoses (DD: 91.1 ± 2.3% with gene features vs. 90.4 ± 2.3% with GO features; ASD: 86 ± 3% vs. 85.7 ± 3%), demonstrating robust performance regardless of feature granularity. While gene features showed marginally higher recall, the minimal differences (< 1%) suggest both approaches are comparably effective for case detection.

Lastly, the serotonergic + dopaminergic models utilized substantially more features than serotonergic-only approaches (gene features: 111 ± 6 vs. 51 ± 3; GO features: 68 ± 2 vs. 20 ± 1), with gene-level features consistently outnumbering GO-term counterparts by approximately twofold in both systems (serotonin-only: 51 ± 3 vs. 20 ± 1; combined: 111 ± 6 vs. 68 ± 2). This pattern reflects: (1) the expanded biological scope of dual neurotransmitter systems, (2) the finer granularity of gene-level representations, and (3) the ability of GO features to capture broader genetic patterns with fewer variables.

## Discussion

In this study, we utilized gene and GO term networks to identify disrupted genes and biological mechanisms related to serotonin neurotransmission in ASD and DD. We found serotonergic convergence of molecular functions and biological processes underlying the genetic heterogeneity. Accordingly, although genetic heterogeneity was larger in ASD when examining biological ontologies, the predominant pattern was a homogeneity of serotonergic terms, which was distinct for each type of diagnosis.

Classifying diagnostic categories enabled us to evaluate the predictive quality of both networks. This approach was further tested in an extended setup, using participants’ serotonergic and dopaminergic imbalances to predict their diagnoses.

### Macroscopic features of genetic networks

Macroscopic analysis of the Diagnosis-Gene network identified three genetic sources disrupted in the DD diagnostic category related to serotonergic mechanisms: *GNAI3*, *FCER1G*, and *OR10J5*.

*GNAI3* encodes a subunit of the G-protein complex activated by serotonergic neurotransmitters^60^, linking it to downstream signalling cascades relevant to neurodevelopment^61^. Its association with striatal serotonergic synapses and synaptic long-term depression suggests a potential contribution to the neurodevelopmental features associated with ASD^62^.

*FCER1G* encodes the gamma subunit of the high-affinity IgE receptor (FcεRI), which mediates immune responses through the release of serotonin and other inflammatory mediators by mast cells and platelets^63–66^. The hyperserotonemia consistently observed in ASD suggests a link between *FCER1G*-mediated immune activation, serotonin metabolism, and neurodevelopmental symptomatology^63^^,^^67^.

*OR10J5* encodes an olfactory receptor expressed not only in the nasal epithelium^68^. but also in gastrointestinal tissues, where its activation enhances serotonin release via intracellular Ca^2+^ signaling. Dysregulated *OR10J5* expression may therefore contribute to the gut-brain serotonergic interactions associated with ASD^69^.

This study showed that it is likely more than one gene disruption is involved in ASD and DD. A similar pattern was also noticed in our previous work, where we studied multiple disruptions in genes related to dopaminergic neurotransmission in ASD and DD^42^. Together, these results support that, in most cases, these types of diseases are highly polygenic and their etiology extremely complex^70^. However, a major distinction of the current work was the identification of convergence of serotonergic biological mechanisms.

Regarding the GO network, macroscopic analysis did indeed identify serotonergic mechanisms coded by frequently disrupted genes for each diagnosis. This analysis showed convergence in four GO terms: three concerning serotonergic receptor activity and one related to serotonin secretion in the bloodstream by platelets. Here, the convergence was strikingly more evident than in our previous work with genes involved in dopamine neurotransmission in participants with ASD and DD^42^, consistent with serotonergic convergence that adds discriminatory value beyond dopaminergic features.

Importantly, this network exposed a convergent pattern of homogeneous serotonergic mechanisms despite genetic heterogeneity. Part of this convergence originated from the interactions of participants with the three gene hubs identified in the Gene network analysis (Table 3). Additionally, even genes that have a small number of links in the Diagnosis-Gene network do target the same serotonergic mechanisms associated with the identified gene hubs.

Thus, this result showed the importance of networks in identifying key biological agents in complex disease settings, particularly when genetic heterogeneity is involved. The literature addresses this subject not only in ASD but also in other neurodevelopmental and complex disorders^71–73^.

### Differences in Network Structures

The centrality measures of the two networks (Table 2) and the analysis of their gene and GO hubs (Table 3) provide a clear picture of the differences between the two network structures. In short, looking at the centrality measures, we detected that the Diagnosis-GO network was more connected than the Diagnosis-Gene network and that the maximum distance between nodes in the Diagnosis-GO network was shorter than in the Diagnosis-Gene network. More precisely, we detected a difference between the diameters of the networks of 6 nodes. These results suggest the existence of communities where nodes are more strongly tied to each other in the Diagnosis-GO network than in the Diagnosis-Gene network, confirming the validity of this approach in identifying biological features.

The existence of network communities is a common feature in biological networks, and the nodes connected in a particular community usually participate in the same biological process^74–79^.

The analysis of the network’s hubs showed that three of the four hubs in the Diagnosis-GO network had more links than the hubs present in the Diagnosis-Gene network (Table 3). These GO hubs targeted the activity of serotonergic receptors and shared links with the identified Gene hubs. The Diagnosis-Gene network (Fig. 3) and the average neighbor degree (Table 3) of the Gene hubs indicate the presence of a subnetwork, where participants in this subnetwork often had a DD diagnosis.

However, these GO hubs have more connections with participants than the Gene hubs, and the participants linked to them have, on average, a link with three other GO terms. This suggests that there might be other genetic subnetworks involved in the disruption of serotonergic receptor activity. To capture them, we used gene generalized similarity networks.

### Contrast between and within diagnoses

The use of link similarities between genes and participants within a particular diagnosis allowed us to isolate genetic subnetworks specific to each diagnosis, as well as their associated serotonergic mechanisms. This technique led to the creation of the ASD Gene-GO Network (Fig. 5) and the DD Gene-GO Network (Fig. 6). These networks highlight clusters of genes frequently linked to participants in each diagnosis, enabling genetic comparisons both between and within diagnoses.

In the ASD Gene-GO network, we identified six different genetic clusters, five of which had at least one gene linked to a GO hub involved in some serotonergic receptor mechanism. One of these clusters included the three gene hubs identified earlier. This network suggests that different groups of participants with ASD share genetic disruptions characterized by one of these subnetworks.

Interestingly, the subnetworks identified here were isolated from each other, implying that these genetic signatures are specific and not shared by participants with ASD in different clusters. Compared to our previous work^42^ on dopamine, this pattern was also observed in participants with ASD but often with a higher number of genes per gene cluster, leading to a less homogeneous set of dopaminergic mechanisms associated with each cluster.

Regarding the serotonergic mechanisms targeted by these genetic subnetworks, in five of the six gene clusters, at least one gene in each cluster affects G protein-coupled serotonin receptor activity. Disruptions in this activity may be associated with aberrant activation of downstream signaling pathways. Notably, each genetic cluster often tied one or more serotonergic mechanisms to a unique pathway, with no links to other clusters, providing clues about the specific pathways affected in each genetic subnetwork.

The composition of G-protein heterotrimers determines which downstream pathways are activated^80–83^. and alterations in genes encoding alpha subunits—present in several ASD clusters—may therefore affect multiple serotonergic signalling cascades simultaneously^81,84,85^.

Thus, using similarity networks can provide insights into potential disruptions in distinct serotonergic signaling pathways caused by gene interactions within a cluster. This approach may help stratify genetically distinct subgroups within ASD, which could be of value for future targeted research.

A more homogeneous pattern was observed for the DD diagnosis, where only two gene clusters were identified, each sharing several serotonergic mechanisms with the other. This suggests that participants with DD are more similar regarding their genes and serotonergic mechanism disruptions, as also found in our previous work on dopaminergic mechanisms.

Comparing gene similarity networks between the two diagnoses allowed us to identify two very similar subnetworks, differing only by a few genes. These subnetworks include the gene hubs identified earlier. In the DD diagnosis, the subnetwork is extended by the presence of the *HTR1F* gene, whereas in the ASD diagnosis, the *HTR1D* and *CHRM3* genes are included instead. This result suggests that imbalances in these gene subnetworks may be associated with distinct patterns of brain region involvement and cellular processes. For example, *HTR1F* is primarily expressed in striatal and thalamic regions^86,87^, where alterations may contribute to the motor coordination issues and sensory processing anomalies common in DD. *HTR1D*, by contrast, is enriched in the caudate and substantia nigra—basal ganglia structures central to motor control and behavioural regulation—where disruption may contribute to the repetitive behaviours and motor abnormalities characteristic of ASD^86,87^.

*CHRM3* is predominantly expressed in the parasympathetic nervous system and in cortical and hippocampal regions, contributing to both inhibitory and excitatory modulation^88^. Dysregulation of *CHRM3* may therefore be associated with the gastrointestinal dysfunction and atypical stress responses frequently reported in ASD^89^.

At the cellular level, the transcription products of *HTR1D*, *HTR1F*, and *CHRM3* genes play a role in adenylate cyclase inhibition, a critical mechanism for regulating cAMP levels within cells. Disruptions in this pathway have been associated with widespread neural dysfunction and may be relevant to the varied clinical features of DD and ASD.

These results illustrate how a systems-level approach can identify biologically coherent patterns within genetically heterogeneous conditions—a pattern also documented in other complex neurodevelopmental and psychiatric disorders^90–92^ and that a substantial fraction of genes may contribute to variation in disease risk.

### Prediction of participants diagnosis with serotonergic and dopaminergic genetic features

The final step of this study aimed to use a fraction of the connections between participants and disrupted genes, or GO terms related to serotonergic and dopaminergic mechanisms, to train a differential diagnosis classification Random Forest algorithm. This methodology allowed us to test the usefulness of the information contained in the Diagnosis-Gene Network (Fig. 3) and the Diagnosis-GO Network (Fig. 4) for classifying between conditions. It also enabled us to verify the robustness, generalization ability, and prevent overfitting of the model.

All network features were computed once on the full bipartite networks to preserve global topological structure and maximize statistical power. This fixed feature space avoids redundant calculations and aligns with best practices in graph-based ML. Prior studies suggest such gene/GO-centric metrics are robust to participant sub-sampling when all gene/GO nodes are retained^93,94^, supporting the reliability of our approach.

Furthermore, we extended this approach by incorporating participants’ genetic imbalances related to dopamine-dependent processes. Previously, we studied participants with ASD and DD, focusing only on dopamine features, and achieved a prediction accuracy in test samples ranging from 83.22% ± 1.09% to 85.18% ± 1.11%, depending on the type of features used (Train size: 1846 participants (923 ASD/923 DD); Test Size: 790 participants (395 ASD/395 DD))^42^. By combining dopamine and serotonin features, we tested the potential of using information on multiple neurotransmitters involved in neurodevelopmental diseases to predict diagnoses. For GO features, combining dopaminergic and serotonergic signals significantly improved classification (*p* = 0.003), with a large effect size (Cohen’s d = 2.02), consistent with the two systems capturing partially non-overlapping biological information. For gene-dosage features, the same direction of effect was observed (Cohen’s d = 0.98, Wilcoxon *p* < 0.001), though the conservative Nadeau & Bengio correction did not reach significance (*p* = 0.14). These results indicate that serotonergic CNVs contribute discriminatory information that, when added to dopaminergic features, significantly improves ASD versus DD classification. Whether this reflects independent causal roles, convergent downstream effects, or correlated pleiotropy cannot be determined from the present data.

In this study, the prediction accuracy of the diagnoses in test samples across different models varied from 82.40% ± 2.1% to 88.6% ± 1.8%. Review studies of classification models attempting to predict the diagnosis between participants with ASD and controls show a wide range of prediction accuracy from 52 to 100%^95–98^. Among the reviewed studies, several different approaches were noted regarding the type of data used (psychometrics, imaging, and genetics), the algorithm of choice (from classic machine learning using linear regression, SVM, and classification trees to more modern approaches using deep learning algorithms), the balance between classes (some used a similar number between classes whereas other studies used very imbalanced samples), and the sample sizes (the majority trained and tested algorithms with fewer than one hundred participants, some managed to gather samples over that number, and a few used more than one thousand participants).

Regarding the type of data used, the large majority of studies employed brain imaging data to make diagnostic predictions^96–98^. Only three studies used genetic data: one used CNVs^99^, and the other two used SNPs (Single-Nucleotide Polymorphisms)^100,101^. Thus, it is important to boost the use of genetic data in attempting to predict the diagnosis of neurodevelopmental disorders. One of the advantages of these approaches is that genetic samples can be collected very early in life or even during gestational periods, which could help in identifying cases requiring special medical attention earlier. Furthermore, the best accuracy achieved among studies using genetic data was 73.67%, which considered SNPs in three genes (a total of ten SNPs)^100^. In this work, we showed that ASD and DD exhibit greater genetic heterogeneity and that using polygenic models or gene ontology-driven models could improve prediction results without losing generalization power.

Additionally, we noticed the same discrepancies in accuracy prediction in both traditional classification algorithms and deep learning algorithms^96,97^. This indicates that other factors might have a more significant impact on classification outcomes. In fact, higher accuracy was often observed in small sample sizes (fewer than one hundred subjects), while lower accuracy was more common in larger sample sizes (around one thousand subjects), suggesting a bias. This observation raises questions about generalization problems in studies with small sample sizes. For example, studies with samples of 27 (11 ASD; 16 Controls), 34 (17 ASD; 17 Controls), and 30 (15 ASD; 15 Controls) achieved classification accuracies of 100%, 97%, and 96.7%, respectively^102–104^ In contrast, studies with samples of 964 (447 ASD; 517 Controls), 1035 (505 ASD; 530 Controls), and 1112 (539 ASD; 573 Controls) participants reported accuracies of 60%, 70%, and 68.5%, respectively^105–107^.

In this study, we aimed to mitigate such issues by using a larger data sample (817 participants: 409 ASD; 408 DD). Moreover, to test generalization and avoid sampling bias, we employed balanced classes in both train and test sets, along with randomization and repetition. This approach helped us better understand and assess the prediction capabilities of our methodology. For example, the serotonergic + dopaminergic models achieved robust recall rates (> 85% for ASD; > 90% for DD), comparable to DSM-5 clinician sensitivity ranges (81–95%) in differential diagnosis contexts^108^. When combined with high positive predictive values (> 90% for ASD; > 86% for DD), these results are consistent with potential utility as a computational adjunct in differential diagnosis research, though prospective clinical validation would be required before any clinical application.

Moreover, for clinical implementation, both serotonergic + dopaminergic feature types performed comparably (DD recall: 90.4–91.1%; ASD recall: 85.7–86%), with gene features showing only minimal advantages (< 1% difference). This suggests GO-term features could be a viable alternative when gene-level data is unavailable—a clinically relevant scenario given the substantial genetic heterogeneity in ASD and DD. While gene-level data may be inconsistent across cohorts due to variant diversity, GO terms abstract this heterogeneity into broader, more homogeneous functional patterns, enabling robust performance with fewer variables. Notably, GO features achieved this parity while using ~ 40% fewer features than gene-level counterparts (68 ± 2 vs. 111 ± 6), suggesting they may offer superior generalizability across genetically divergent populations without sacrificing diagnostic accuracy.

### Pitfalls and future work

To conclude, we address study limitations and future directions. In this work, we used CNV data, which is typically harvested from blood or saliva and detects genetic variations of more than 50 k base pairs. Additionally, the CNVs used in this study were collected from genetic studies aimed at estimating the relative contribution of a single genetic variation in defining the variation in the trait of study across a population. Consequently, interactions of genes present in CNVs with insufficient significant power, as well as interactions of genes present in common variants, could not be studied here.

The 249 unique CNVs in the serotonergic cohort had a median size of 48.3 Mb (IQR: 1.2–131.2 Mb; range: 1.2 kb–231.9 Mb), with 77.5% exceeding 1 Mb in length. Given these large sizes, many CNVs span multiple genes: 60.7% of CNVs (n = 151) overlapped a single serotonergic gene, while 39.3% (n = 98) overlapped two or more (range 2–6). When a gene was overlapped by more than one CNV, it was counted once per participant, preventing inflation of feature values. Nevertheless, a single large CNV may affect multiple serotonergic genes simultaneously, introducing feature correlation that reflects physical genomic co-localisation rather than independent biological events. Future analyses should consider weighting features by CNV independence or applying methods adapted for structural variants that account for genomic architecture. (Supplementary Tables S2, S3, and S5).

Population allele frequency thresholds and variant transmission data (transmitted vs. de novo) were not uniformly available across contributing studies. Within the serotonergic cohort, transmission status was available for only 13.4% of CNV–patient pairs (6.8% maternal, 6.3% paternal, 0.4% biparental), with the remaining 86.6% recorded as unknown—a proportion too low to use as a covariate or inclusion criterion. Future work should use datasets with uniform variant annotation.

CNVs in the SFARI Gene database are drawn from clinical and research studies in which rarity filters were typically applied, reducing the likelihood of including common benign structural variants. Nevertheless, the ancestry composition of the variant catalogue was not assessed; cross-referencing with gnomAD-SV in future work would allow evaluation of potential ancestry-related biases in the serotonergic gene and GO-term networks.

The results obtained reflect outcomes from a specific genetic subpopulation (SFARI Gene cohort) with balanced ASD/DD prevalence. Further validation in more heterogeneous populations—including diverse ancestries, comorbid conditions, and clinical settings—is needed to confirm generalizability and optimize real-world implementation.

Formal sub-sampling analyses were not applied to the bipartite networks. Classifier stability is supported by low variance across 100 random train-test splits (SD ≤ 2.2%). For network topology, prior work suggests that gene-centric metrics are robust to random participant sub-sampling when gene nodes are fully retained (Poisot et al., 2016; Stumpf et al., 2008); participant-level metrics may be more sensitive, and this is acknowledged as a limitation.

In future studies, we would like to explore interactions that go beyond the genetic variations captured by CNVs to better model the genetic state of an individual. This could help to more closely capture the genetic serotonergic state of an individual and identify patterns that could clarify associations between genetic variation and neurodevelopmental features. Another interesting approach will be to explore these genetic variations and their interactions with brain anatomy and function. Ultimately, it would be desirable to link genetics and the brain to understand associations with behavior.

## Conclusion

In this study, we explored the potential of complex network analysis and machine learning to differentiate between ASD and DD by examining CNVs in serotonergic coding genes. Although our findings underscore the genetic heterogeneity in ASD and the relative homogeneity in DD, we found a surprising convergence of serotonergic mechanisms, which was distinct across diseases.

By constructing and analyzing diagnosis-gene and diagnosis-GO term networks, we identified key serotonergic mechanisms and genetic clusters specific to each condition. ASD was characterized by six distinct genetic clusters, revealing a diverse genetic landscape, which converged to a small set of serotonergic mechanisms, while DD exhibited two main clusters, suggesting a more uniform genetic basis. The convergence of unique and overlapping serotonergic mechanisms in both conditions is consistent with serotonergic CNVs contributing discriminatory information that is partially non-overlapping with dopaminergic features.

Our machine learning models demonstrated significant predictive power, achieving an accuracy of 85.6% using serotonergic gene features alone, and 88.6% when combining serotonergic and dopaminergic features. These results demonstrate the potential of integrating genetic network features for classification of NNDs in a research setting.

The study also emphasized the importance of considering both individual genetic variations and their broader biological contexts. The network analysis revealed that despite the genetic heterogeneity in ASD, there is a convergence of disrupted serotonergic mechanisms, suggesting common biological pathways that may warrant further mechanistic investigation.

Future research should aim to further explore the interplay between serotonergic and dopaminergic systems and their associations with neurodevelopmental outcomes. Additionally, integrating genetic data with brain imaging and functional studies could provide a more comprehensive understanding of how these genetic variations influence neural circuitry and behavior.

## Supplementary Information

Below is the link to the electronic supplementary material.

Supplementary Material 1

## Data Availability

The raw data as well as the data supporting reported results can be found in this repository: A.S., F.C., J.B.M., M.C.-B. Study repository: A relational database of SFARI Gene CNVs data integrated with associated genes and GO terms for the study of genetics in neurodevelopmental disorders—Autism Imaging Genetics Dataverse (Internet). 2022. Available online: 10.7910/DVN/HO1JLJ.

## Abbreviations

5-HT: Serotonin (5-Hydroxytryptamine)
5-HTR: Serotonin receptor
ACMG: American College of Medical Genetics and Genomics
ADHD: Attention deficit hyperactivity disorder
ASD: Autism spectrum disorder
AUC: Area under the curve
ClinGen: Clinical genome resource
CMA: Chromosomal microarray analysis
CNS: Central nervous system
CNV: Copy number variation
DA: Dopamine
DD: Developmental delay
FN: False negatives
FP: False positives
FR: Fruchterman–Reingold (algorithm)
GO: Gene ontology
ML: Machine learning
NAc: Nucleus accumbens
NDD: Neurodevelopmental disease
PNS: Peripheral nervous system
SFARI: Simons Foundation Autism Research Initiative
SNP: Single-nucleotide polymorphism
SNc: Substantia Nigra pars compacta
SSC: Simons simplex collection
TN: True negatives
TP: True positives
VTA: Ventral tegmental area
