# Supplementary material for: Network analysis of serotonin CNVs shows biological convergence from genetic heterogeneity and discriminates between autism and developmental delay
Source: Sci Rep. 2026 May 26;16:23977. doi: 10.1038/s41598-026-54264-y (PMC13434470; doi:10.1038/s41598-026-54264-y)
Supplement: Supplementary file 1 [file 41598_2026_54264_MOESM1_ESM.docx]

**Supplementary Information**

# Supplementary Tables

**Table S1. Participant demographics by diagnosis group.**

| **Diagnosis** | **N** | **Male** | **Female** | **Sex unknown** | **Age (mean ± SD)** |
| --- | --- | --- | --- | --- | --- |
| ASD | 409 | 165 (40.3%) | 32 (7.8%) | 212 (51.8%) | 10.9 ± 4.8 (n=100) |
| DD | 408 | 25 (6.1%) | 26 (6.4%) | 357 (87.5%) | Not available |

Note. Sex unknown includes participants recorded as ‘u’ or ‘unk’ in the database. Age at diagnosis was available for 100/409 ASD participants (range 4–31 years) and for none of the DD participants.

**Table S1b. Family profile by diagnosis group.**

| **Diagnosis** | **N** | **Simplex** | **Multiplex** | **Unknown** |
| --- | --- | --- | --- | --- |
| ASD | 409 | 165 (40.3%) | 38 (9.3%) | 206 (50.4%) |
| DD | 408 | 4 (1.0%) | 3 (0.7%) | 401 (98.3%) |

Note. Family profile (simplex vs. multiplex) was available for 49.6% of ASD and fewer than 2% of DD participants.

**Table S2. CNV size distribution statistics (serotonergic cohort).**

| **Statistic** | **Value** |
| --- | --- |
| Unique CNVs | 249 |
| CNV–patient pairs | 842 |
| Median size | 48.33 Mb |
| Mean size | 68.59 Mb |
| 25th percentile | 1.19 Mb |
| 75th percentile | 131.24 Mb |
| IQR | 130.05 Mb |
| Minimum | 1,183 bp (0.001 Mb) |
| Maximum | 231.90 Mb |

Note. CNV type breakdown: 55.7% duplications, 44.3% deletions. Unknown and complex CNV types excluded.

**Table S3. Distribution of unique CNVs by size category.**

| **Size category** | **N (unique CNVs)** | **%** |
| --- | --- | --- |
| < 100 kb | 26 | 10.4% |
| 100 kb – 1 Mb | 30 | 12.0% |
| > 1 Mb | 193 | 77.5% |

**Table S4. CNV transmission/inheritance status in the serotonergic cohort.**

| **Inheritance** | **N (CNV–patient pairs)** | **%** |
| --- | --- | --- |
| Unknown | 729 | 86.58% |
| Maternal | 57 | 6.77% |
| Paternal | 53 | 6.29% |
| Both (biparental) | 3 | 0.36% |
| Total | 842 | 100% |

Note. Transmission status was available for only 13.4% of CNV–patient pairs, precluding its use as a covariate.

**Table S5. Number of serotonergic genes overlapped per unique CNV.**

| **Serotonergic genes per CNV** | **N (unique CNVs)** | **%** |
| --- | --- | --- |
| 1 | 151 | 60.64% |
| 2 | 36 | 14.46% |
| 3 | 28 | 11.24% |
| 4 | 11 | 4.42% |
| 5 | 22 | 8.84% |
| 6 | 1 | 0.40% |
| Total | 249 | 100% |

Note. When a CNV overlapped multiple serotonergic genes, each gene was counted once per participant. No double-counting across CNVs.

**Table S6. Random Forest classification performance: serotonin-only vs. combined (serotonin + dopamine) models.**

| **Feature type** | **Serotonin-only** | **Combined** | **Δ** | **NB t(99) / p** | **Cohen’s d** | **Wilcoxon W / p** |
| --- | --- | --- | --- | --- | --- | --- |
| Gene-dosage | 85.6 ± 2.2% | 88.6 ± 1.8% | +3.0% | 1.48 / .142 | 0.98 | 356.0 / < .001 |
| GO terms | 82.4 ± 2.1% | 88.0 ± 1.8% | +5.6% | 3.05 / .003 | 2.02 | 15.0 / < .001 |

Note. Serotonin-only and combined models were run on the same cohort (409 ASD / 408 DD) using 100 random 70/30 train–test splits. Statistical comparison used the Nadeau & Bengio corrected resampled t-test (NB) and Wilcoxon signed-rank test. Descriptive reference: the previously published dopaminergic model (1,318/1,327 cohort) achieved 85.6 ± 1.1% (gene) and 82.0 ± 1.3% (GO).

**Table S7. Extended topological metrics for serotonergic bipartite networks (ASD vs. DD).**

| **Metric** | **Gene-dosage ASD** | **Gene-dosage DD** | **GO ASD** | **GO DD** |
| --- | --- | --- | --- | --- |
| Participants | 353 | 356 | 409 | 408 |
| Active feature nodes | 30 genes | 23 genes | 20 GO terms | 20 GO terms |
| Edges | 819 | 929 | 1,290 | 1,333 |
| Average clustering coefficient | 0.676 | 0.924 | 0.486 | 0.672 |
| Global efficiency | 0.263 | 0.276 | 0.413 | 0.424 |
| Modularity (Louvain) | 0.610 | 0.170 | 0.237 | 0.208 |
| Communities detected | 7 | 9 | 3 | 3 |

Note. Gene-dosage participant counts (353/356) are lower than the full cohort (409/408) because participants with no gene-level serotonergic CNV features were excluded from the gene network.

**Table S8. Top hub genes by degree and betweenness centrality in the gene-dosage bipartite network.**

| **Rank** | **ASD gene (DC)** | **ASD DC** | **DD gene (DC)** | **DD DC** | **ASD gene (BC)** | **ASD BC** | **DD gene (BC)** | **DD BC** |
| --- | --- | --- | --- | --- | --- | --- | --- | --- |
| 1 | HTR7 | 0.258 | GNAI3 | 0.747 | HTR7 | 0.421 | GNAI3 | 0.196 |
| 2 | GNAI1 | 0.227 | FCER1G | 0.725 | GNAI1 | 0.217 | FCER1G | 0.161 |
| 3 | DDC | 0.196 | OR10J5 | 0.725 | GNAI3 | 0.202 | OR10J5 | 0.161 |
| 4 | GNAI3 | 0.193 | GNAZ | 0.135 | OR11H4 | 0.183 | GNAZ | 0.016 |
| 5 | CHRM2 | 0.153 | GNAI1 | 0.051 | DDC | 0.163 | HTR1F | 0.004 |

Note. DC = degree centrality; BC = betweenness centrality. Values are normalised and range from 0 to 1.

**Table S9. Top hub GO terms by degree and betweenness centrality in the GO-term bipartite network.**

| **Rank** | **ASD GO term** | **DC** | **DD GO term** | **DC** |
| --- | --- | --- | --- | --- |
| 1 | G protein-coupled serotonin receptor activity (GO:0004993) | 0.738 | G protein-coupled serotonin receptor binding (GO:0031821) | 0.814 |
| 2 | G protein-coupled serotonin receptor signaling pathway (GO:0098664) | 0.653 | G protein-coupled serotonin receptor activity (GO:0004993) | 0.743 |
| 3 | G protein-coupled serotonin receptor binding (GO:0031821) | 0.389 | G protein-coupled serotonin receptor signaling pathway (GO:0098664) | 0.721 |
| 4 | Serotonin binding (GO:0051378) | 0.220 | Serotonin secretion by platelet (GO:0002554) | 0.645 |
| 5 | Serotonin biosynthetic process (GO:0042427) | 0.198 | Aralkylamine N-acetyltransferase activity (GO:0004059) | 0.054 |

Note. Betweenness centrality rankings mirror degree centrality for both networks.

**Table S10. Curated GO terms for serotonergic neurotransmission (n = 59).**

| **GO ID** | **Name** | **Aspect** | **Definition (abbreviated)** |
| --- | --- | --- | --- |
| GO:0006837 | serotonin transport | biological process | The directed movement of serotonin into, out of or within a cell, or between cells, by means of some agent such as a transporter or pore. |
| GO:0051610 | serotonin uptake | biological process | The directed movement of serotonin into a cell, typically presynaptic neurons or glial cells. |
| GO:0051378 | serotonin binding | molecular function | Binding to serotonin (5-hydroxytryptamine), a monoamine neurotransmitter occurring in the peripheral and central nervous systems. |
| GO:0001820 | serotonin secretion | biological process | The regulated release of serotonin by a cell. |
| GO:0042428 | serotonin metabolic process | biological process | The chemical reactions and pathways involving serotonin (5-hydroxytryptamine). |
| GO:0099589 | serotonin receptor activity | molecular function | Combining with the biogenic amine serotonin and transmitting a signal across a membrane by activating some effector activity. |
| GO:1904014 | response to serotonin | biological process | Any process that results in a change in state or activity of a cell or an organism as a result of a serotonin stimulus. |
| GO:0042427 | serotonin biosynthetic process | biological process | The chemical reactions and pathways resulting in the formation of serotonin (5-hydroxytryptamine). |
| GO:0042429 | serotonin catabolic process | biological process | The chemical reactions and pathways resulting in the breakdown of serotonin (5-hydroxytryptamine). |
| GO:0098665 | serotonin receptor complex | cellular component | A protein complex that is capable of serotonin receptor activity. |
| GO:0060096 | serotonin secretion, neurotransmission | biological process | The regulated release of serotonin by a cell, in which released serotonin acts as a neurotransmitter. |
| GO:0051614 | inhibition of serotonin uptake | biological process | Any process that prevents the activation of the directed movement of serotonin into a cell. |
| GO:0051611 | regulation of serotonin uptake | biological process | Any process that modulates the frequency, rate or extent of the directed movement of serotonin into a cell. |
| GO:0002554 | serotonin secretion by platelet | biological process | The regulated release of serotonin by a platelet or group of platelets. |
| GO:0002556 | serotonin secretion by basophil | biological process | The regulated release of serotonin by a basophil or group of basophils. |
| GO:0014062 | regulation of serotonin secretion | biological process | Any process that modulates the frequency, rate or extent of the regulated release of serotonin. |
| GO:1904015 | cellular response to serotonin | biological process | Any process that results in a change in state or activity of a cell as a result of a serotonin stimulus. |
| GO:0007210 | serotonin receptor signaling pathway | biological process | The series of molecular signals generated as a consequence of a serotonin receptor binding to one of its physiological ligands. |
| GO:0031833 | type 7 serotonin receptor binding | molecular function | Binding to a type 7 serotonin receptor. |
| GO:0031831 | type 5B serotonin receptor binding | molecular function | Binding to a type 5B serotonin receptor. |
| GO:0031832 | type 6 serotonin receptor binding | molecular function | Binding to a type 6 serotonin receptor. |
| GO:0031830 | type 5A serotonin receptor binding | molecular function | Binding to a type 5A serotonin receptor. |
| GO:0031824 | type 1E serotonin receptor binding | molecular function | Binding to a type 1E serotonin receptor. |
| GO:0031825 | type 1F serotonin receptor binding | molecular function | Binding to a type 1F serotonin receptor. |
| GO:0031822 | type 1B serotonin receptor binding | molecular function | Binding to a type 1B serotonin receptor. |
| GO:0031823 | type 1D serotonin receptor binding | molecular function | Binding to a type 1D serotonin receptor. |
| GO:0031828 | type 2C serotonin receptor binding | molecular function | Binding to a type 2C serotonin receptor. |
| GO:0031829 | type 4 serotonin receptor binding | molecular function | Binding to a type 4 serotonin receptor. |
| GO:0031826 | type 2A serotonin receptor binding | molecular function | Binding to a type 2A serotonin receptor. |
| GO:0031827 | type 2B serotonin receptor binding | molecular function | Binding to a type 2B serotonin receptor. |
| GO:0006587 | serotonin biosynthetic process from tryptophan | biological process | The chemical reactions and pathways resulting in the formation from tryptophan of serotonin (5-hydroxytryptamine). |
| GO:0051613 | positive regulation of serotonin uptake | biological process | Any process that activates or increases the frequency, rate or extent of the directed movement of serotonin into a cell. |
| GO:0051612 | negative regulation of serotonin uptake | biological process | Any process that stops, prevents, or reduces the frequency, rate or extent of the directed movement of serotonin into a cell. |
| GO:0002552 | serotonin secretion by mast cell | biological process | The regulated release of serotonin by a mast cell or group of mast cells. |
| GO:1905627 | regulation of serotonin biosynthetic process | biological process | Any process that modulates the frequency, rate or extent of serotonin biosynthetic process. |
| GO:0014064 | positive regulation of serotonin secretion | biological process | Any process that activates or increases the frequency, rate or extent of the regulated release of serotonin. |
| GO:0014063 | negative regulation of serotonin secretion | biological process | Any process that stops, prevents, or reduces the frequency, rate or extent of the regulated release of serotonin. |
| GO:0160039 | serotonin-gated chloride channel activity | molecular function | Enables the transmembrane transfer of a chloride ion by a channel that opens when serotonin has been bound. |
| GO:0031821 | G protein-coupled serotonin receptor binding | molecular function | Binding to a metabotropic serotonin receptor. |
| GO:0002442 | serotonin secretion involved in inflammatory response | biological process | The regulated release of serotonin by a cell as part of an inflammatory response. |
| GO:1905629 | positive regulation of serotonin biosynthetic process | biological process | Any process that activates or increases the frequency, rate or extent of serotonin biosynthetic process. |
| GO:1905628 | negative regulation of serotonin biosynthetic process | biological process | Any process that stops, prevents or reduces the frequency, rate or extent of serotonin biosynthetic process. |
| GO:0002351 | serotonin production involved in inflammatory response | biological process | The synthesis or release of serotonin following a stimulus as part of an inflammatory response. |
| GO:0004993 | G protein-coupled serotonin receptor activity | molecular function | Combining with the biogenic amine serotonin and transmitting the signal across the membrane by activating an associated G-protein. |
| GO:0098666 | G protein-coupled serotonin receptor complex | cellular component | A protein complex that is capable of G protein-coupled serotonin receptor activity. |
| GO:0001586 | Gi/o-coupled serotonin receptor activity | molecular function | Combining with serotonin and transmitting the signal across the membrane by activation of the Gi/o subunit of an associated G-protein complex. |
| GO:0001587 | Gq/11-coupled serotonin receptor activity | molecular function | Combining with serotonin and transmitting the signal across the membrane by activation of the Gq/11 subunit of an associated G-protein complex. |
| GO:1904602 | serotonin-activated cation-selective channel complex | cellular component | A protein complex capable of serotonin-activated cation-selective channel activity. |
| GO:0140227 | serotonin-gated cation-selective signaling pathway | biological process | The series of molecular signals initiated by serotonin binding to a serotonin receptor, followed by movement of ions through a channel in the receptor complex. |
| GO:0022850 | serotonin-gated monoatomic cation channel activity | molecular function | Enables the transmembrane transfer of a cation by a channel that opens when serotonin has been bound. |
| GO:0098664 | G protein-coupled serotonin receptor signaling pathway | biological process | The series of molecular signals generated as a consequence of a G protein-coupled serotonin receptor binding to one of its physiological ligands. |
| GO:0007208 | phospholipase C-activating serotonin receptor signaling pathway | biological process | A signaling pathway initiated by serotonin binding to its receptor, activating phospholipase C. |
| GO:0007192 | adenylate cyclase-activating serotonin receptor signaling pathway | biological process | An adenylate cyclase-activating G protein-coupled receptor signaling pathway initiated by serotonin. |
| GO:0007198 | adenylate cyclase-inhibiting serotonin receptor signaling pathway | biological process | An adenylate cyclase-inhibiting G protein-coupled receptor signaling pathway initiated by serotonin. |
| GO:0014076 | response to fluoxetine | biological process | Any process that results in a change in state or activity of a cell or an organism as a result of a fluoxetine stimulus. |
| GO:0140004 | histone H3Q5ser reader activity | molecular function | A histone reader that recognizes a histone H3 serotonylated at glutamine 5. |
| GO:1904123 | positive regulation of fatty acid beta-oxidation by serotonin receptor signaling pathway | biological process | A serotonin receptor signaling pathway that results in positive regulation of fatty acid beta-oxidation. |
| GO:0005335 | serotonin:sodium:chloride symporter activity | molecular function | Enables the transfer of serotonin according to the reaction: serotonin(out) + Na+(out) + Cl-(out) = serotonin(in) + Na+(in) + Cl-(in). |
| GO:0004059 | aralkylamine N-acetyltransferase activity | molecular function | Catalysis of the reaction: acetyl-CoA + an aralkylamine = CoA + an N-acetylaralkylamine. |

Note. GO terms curated from the QuickGO API and used to filter serotonergic genes from the SFARI Gene CNV module. Aspect: BP = biological process; MF = molecular function; CC = cellular component.

**Table S11. Serotonergic genes identified through GO term annotation in the SFARI Gene CNV module (n = 60).**

| **Ensembl ID** | **Gene Name** | **GO IDs** |
| --- | --- | --- |
| ENSG00000129673 | AANAT | GO:0004059 |
| ENSG00000186723 | AC114267.1 | GO:0004993, GO:0098664 |
| ENSG00000141480 | ARRB2 | GO:0031826 |
| ENSG00000165240 | ATP7A | GO:0042428 |
| ENSG00000183826 | BTBD9 | GO:0042428 |
| ENSG00000168539 | CHRM1 | GO:0004993, GO:0098664 |
| ENSG00000181072 | CHRM2 | GO:0004993, GO:0098664 |
| ENSG00000133019 | CHRM3 | GO:0004993, GO:0098664 |
| ENSG00000180720 | CHRM4 | GO:0004993, GO:0098664 |
| ENSG00000184984 | CHRM5 | GO:0004993, GO:0098664 |
| ENSG00000132437 | DDC | GO:0042427 |
| ENSG00000069696 | DRD4 | GO:0004993, GO:0098664 |
| ENSG00000158869 | FCER1G | GO:0002554 |
| ENSG00000088256 | GNA11 | GO:0031826 |
| ENSG00000127955 | GNAI1 | GO:0031821 |
| ENSG00000065135 | GNAI3 | GO:0031821 |
| ENSG00000156052 | GNAQ | GO:0031826 |
| ENSG00000128266 | GNAZ | GO:0031821 |
| ENSG00000046653 | GPM6B | GO:0051612 |
| ENSG00000183454 | GRIN2A | GO:0042428 |
| ENSG00000196639 | HRH1 | GO:0004993, GO:0098664 |
| ENSG00000113749 | HRH2 | GO:0004993, GO:0098664 |
| ENSG00000101180 | HRH3 | GO:0004993, GO:0098664, GO:0014063 |
| ENSG00000134489 | HRH4 | GO:0004993, GO:0098664 |
| ENSG00000178394 | HTR1A | GO:0004993, GO:0098664, GO:0007210, GO:0051378, GO:0042428, GO:0007198, GO:0014062 |
| ENSG00000179546 | HTR1D | GO:0004993, GO:0098664 |
| ENSG00000179097 | HTR1F | GO:0004993, GO:0098664, GO:0051378 |
| ENSG00000102468 | HTR2A | GO:0004993, GO:0098664, GO:0007210, GO:0051378, GO:0007208 |
| ENSG00000135914 | HTR2B | GO:0004993, GO:0098664, GO:0007210, GO:0051378 |
| ENSG00000147246 | HTR2C | GO:0004993, GO:0007210, GO:0051378, GO:0007208, GO:0001587 |
| ENSG00000166736 | HTR3A | GO:0007210, GO:0051378, GO:0022850, GO:1904602 |
| ENSG00000149305 | HTR3B | GO:0007210, GO:0022850, GO:1904602 |
| ENSG00000178084 | HTR3C | GO:0007210, GO:0022850 |
| ENSG00000186090 | HTR3D | GO:0007210, GO:0022850 |
| ENSG00000186038 | HTR3E | GO:0007210, GO:0022850 |
| ENSG00000164270 | HTR4 | GO:0004993, GO:0098664 |
| ENSG00000157219 | HTR5A | GO:0004993, GO:0098664, GO:0051378, GO:0007198 |
| ENSG00000158748 | HTR6 | GO:0004993, GO:0098664 |
| ENSG00000148680 | HTR7 | GO:0004993, GO:0098664 |
| ENSG00000069535 | MAOB | GO:0014063 |
| ENSG00000089250 | NOS1 | GO:0051612 |
| ENSG00000171942 | OR10H2 | GO:0004993, GO:0098664 |
| ENSG00000171936 | OR10H3 | GO:0004993, GO:0098664 |
| ENSG00000176231 | OR10H4 | GO:0004993, GO:0098664 |
| ENSG00000172519 | OR10H5 | GO:0004993, GO:0098664 |
| ENSG00000184155 | OR10J5 | GO:0004993, GO:0098664 |
| ENSG00000176198 | OR11H4 | GO:0004993, GO:0098664 |
| ENSG00000181718 | OR5T2 | GO:0004993, GO:0098664 |
| ENSG00000172489 | OR5T3 | GO:0004993, GO:0098664 |
| ENSG00000181499 | OR6T1 | GO:0004993, GO:0098664 |
| ENSG00000108405 | P2RX1 | GO:0002554 |
| ENSG00000123360 | PDE1B | GO:0042428 |
| ENSG00000164197 | RNF180 | GO:0042428 |
| ENSG00000036565 | SLC18A1 | GO:0051610, GO:0005335 |
| ENSG00000165646 | SLC18A2 | GO:0051610, GO:0005335 |
| ENSG00000145335 | SNCA | GO:0051612 |
| ENSG00000145545 | SRD5A1 | GO:0042428 |
| ENSG00000165025 | SYK | GO:0002554, GO:0001820 |
| ENSG00000129167 | TPH1 | GO:0042427 |
| ENSG00000139287 | TPH2 | GO:0042427 |

# Supplementary Discussion

## 4.1. Macroscopic Features of Genetic Networks

**GNAI3**

The GNAI3 gene encodes a subunit of the G-protein complex, which is activated by coupling of hormones or neurotransmitters resulting in a cascading signalling leading to changes in gene expression and signalling pathways. Melatonin (synthesised from serotonin) is an important neurohormone in this context [60]. Recently, this gene was linked with synaptic long-term depression with a potential link with ASD [61]. Additionally, protein interactomes of striatum-expressed genes involving the protein encoded by the GNAI3 gene and its relationship with serotonergic synapses point to the potential involvement of these alterations in the neurodevelopmental features associated with anxiety disorders [62]. Given the relevance of dopamine in this system, this points to a convergence of serotonergic and dopaminergic mechanisms.

**FCER1G**

The FCER1G gene encodes the gamma subunit of the high-affinity IgE receptor (FcεRI), a protein central to immune responses, where it mediates allergic reactions by triggering the release of inflammatory mediators like histamine, serotonin, and prostaglandins [63]. These mediators interact with the nervous system, altering sensory and autonomic nerve activity and increasing neuronal excitability and synaptic transmission, which can amplify responses to stimuli and cause long-term changes in neuronal function [64]. Serotonin, released during immune activation by mast cells and platelets, plays a significant role in these neuroimmune interactions, influencing both presynaptic and postsynaptic mechanisms and contributing to hypersensitivity in the nervous system [64, 65, 66]. Altered platelet serotonin levels, frequently observed in ASD as hyperserotonemia, suggest a link between immune activation, serotonin metabolism, and neurodevelopmental symptoms such as social and communication deficits [67]. Although the precise role of FCER1G in serotonergic pathways remains unclear, its involvement in regulating serotonin release and immune signalling highlights its potential contribution to ASD and related conditions. The interplay between immune activation and nervous system modulation underscores the broader complexity of neuroimmune interactions, where mast cell activation drives both allergic inflammation and lasting changes in neuronal function, with potential implications for neurodevelopmental and allergic disorders.

**OR10J5**

The OR10J5 gene encodes the olfactory receptor 10J5 protein. Olfactory receptors (OR) are usually expressed in the olfactory epithelium and are mainly responsible for detecting odour molecules, initiating a signalling cascade that ultimately leads to the activation of the olfactory sensory neuron and odour perception. However, the expression of OR has been detected in several extra-nasal tissues, where they participate in the regulation of divergent physiological processes such as cell–cell recognition, pathfinding, and cell cycle processes [68]. In the human gastrointestinal system, known OR ligands can increase intracellular Ca²⁺ concentration, which results in enhanced serotonin release [69], an important regulator of gut peristalsis and secretion. Thus, dysregulated OR expression may significantly contribute to abnormal bowel functions and gut–brain interactions.

## 4.3. Contrast Between and Within Diagnoses

**G-protein heterotrimers**

The composition of G-protein heterotrimers plays a crucial role in cellular functioning, although only a few have been extensively studied [80]. This composition determines which pathways are activated [81] and can potentially activate multiple pathways simultaneously [82]. Different G-proteins can couple to the same serotonin receptor type, leading to the activation of distinct pathways [83]. Additionally, ligand–receptor binding affinity influences G-protein affinity and vice versa, directly impacting the effectiveness of molecular functions [83, 84]. The G-protein complex can also modulate receptor activity, sometimes switching it from active to inactive even in the absence of the agonist [84, 85].

**HTR1F, HTR1D, and CHRM3**

Comparing gene similarity networks between the two diagnoses revealed two very similar subnetworks differing only by a few genes. In the DD diagnosis, the subnetwork is extended by the presence of the HTR1F gene, whereas in the ASD diagnosis, the HTR1D and CHRM3 genes are included instead. This finding suggests that imbalances in these gene subnetworks may be associated with distinct patterns of brain region involvement and cellular processes. For example, HTR1F is primarily expressed in the cortex, dorsal raphe nucleus, hippocampus, hypothalamus, mesentery, striatum, and thalamus [86, 87], while HTR1D is expressed in the caudate, dorsal raphe nucleus, globus pallidus, heart, substantia nigra, intestinal muscle, and at lower levels in the cortex and hippocampus [86, 87].

Alterations in HTR1F expression in the striatum are associated with motor control and sensory processing; disruptions here may contribute to motor coordination issues and sensory processing anomalies common in DD. The caudate and substantia nigra are part of the basal ganglia, which play a crucial role in motor control and behavioural regulation; alterations in HTR1D expression may contribute to the repetitive behaviours and motor abnormalities often associated with ASD.

CHRM3 is predominantly expressed in the parasympathetic nervous system, contributing to both inhibitory and excitatory effects [88]. Dysregulation here may be associated with some of the autonomic dysfunctions observed in ASD, such as gastrointestinal issues or atypical stress responses. In the brain, M3 receptors are mainly expressed in the cortex and hippocampus, with lower levels in the basal forebrain and striatum [89]. Alterations in CHRM3 expression in these regions may be associated with cognitive processes and memory, potentially relevant to the cognitive features observed in ASD.

## Systems Medicine Perspective and Polygenic Complexity

The results presented in this work demonstrate the potential of understanding complex genetic models from a systems medicine perspective to explain differences between and within neurodevelopmental disorders. Some studies have shown that the manifestation of certain complex traits and diseases has a genetic origin widely scattered across the genome [90, 91, 92], and that a substantial fraction of genes may contribute to variation in disease risk.

Moreover, complex trait variants may not be fully explained by genes belonging solely to specific biologically relevant pathways. They can also be influenced by genes not directly linked to these pathways, since cell regulatory networks are highly interconnected. This interconnectedness implies that any expressed gene is likely to affect the regulation or function of relevant pathways in a complex trait [92].
